# Quantifying sleep architecture dynamics and individual differences using big data and Bayesian networks

**DOI:** 10.1371/journal.pone.0194604

**Published:** 2018-04-11

**Authors:** Benjamin D. Yetton, Elizabeth A. McDevitt, Nicola Cellini, Christian Shelton, Sara C. Mednick

**Affiliations:** 1 Department of Psychology, University of California, Irvine, Irvine, California, United States of America; 2 Princeton Neuroscience Institute, Princeton University, Princeton, New Jersey, United States of America; 3 Department of General Psychology, University of Padova, Padova, Italy; 4 Department of Computer Science, University of California, Riverside, Riverside, California, United States of America; Universita degli Studi di Bologna, ITALY

## Abstract

The pattern of sleep stages across a night (sleep architecture) is influenced by biological, behavioral, and clinical variables. However, traditional measures of sleep architecture such as stage proportions, fail to capture sleep dynamics. Here we quantify the impact of individual differences on the dynamics of sleep architecture and determine which factors or set of factors best predict the next sleep stage from current stage information. We investigated the influence of age, sex, body mass index, time of day, and sleep time on static (e.g. minutes in stage, sleep efficiency) and dynamic measures of sleep architecture (e.g. transition probabilities and stage duration distributions) using a large dataset of 3202 nights from a non-clinical population. Multi-level regressions show that sex effects duration of all Non-Rapid Eye Movement (NREM) stages, and age has a curvilinear relationship for Wake After Sleep Onset (WASO) and slow wave sleep (SWS) minutes. Bayesian network modeling reveals sleep architecture depends on time of day, total sleep time, age and sex, but not BMI. Older adults, and particularly males, have shorter bouts (more fragmentation) of Stage 2, SWS, and they transition less frequently to these stages. Additionally, we showed that the next sleep stage and its duration can be optimally predicted by the prior 2 stages and age. Our results demonstrate the potential benefit of big data and Bayesian network approaches in quantifying static and dynamic architecture of normal sleep.

## Introduction

Sleep is a dynamic, multi-dimensional process that reflects lifespan developmental changes in physical and mental health, as well as day-to-day state fluctuations. Alterations in stage patterns and durations (i.e., sleep architecture), are seen in insomnia[1], narcolepsy[2], sleep apnea[3] as well as depression[4] and schizophrenia[5]. However, not all deviations from prototypical sleep are indicators of pathology; individual factors such as age[6], Body Mass Index (BMI)[7], and sex[8] contribute to sleep architecture, and differences are also reported after sleep deprivation[9] or drug use (such as caffeine[10], nicotine[11], alcohol and marijuana[12]). Quantifying the typical variability in sleep architecture and its relation to benign factors, as opposed to those which may be indicative of illness, is of clinical relevance.

Sleep is classically partitioned into 4 stages: Stage 1, Stage 2, Slow Wave Sleep (SWS) and Rapid Eye Movement (REM)[13]. These stages are purported to progress in a specific order across a period of sleep, beginning with Wake → Stage 1, then transitioning into a cyclic repetition of Stage 2 → SWS → Stage 2 → REM[14] (i.e. Ultradian cycles). Additionally, the proportion of time spent in each sleep stage across a night of sleep has been well-defined. The durations of Stage 1 and Stage 2 are relatively constant throughout the night, whereas duration of REM and SWS exhibit a time-dependence, with more SWS at the beginning of the night and a larger proportion of REM in the morning. Throughout the night, sleep is often interrupted with brief bouts of wake, known as Wake After Sleep Onset (WASO). The detection of sleep stages and WASO from the raw Polysomnography signal (PSG) generally follows the Rechtschaffen and Kales (R&K)[15] or American Academy of Sleep Medicine (AASM)[16] guidelines. Criteria differ subtly, where with the AASM criteria there is increased scoring of WASO, Stage 1 and SWS, and decrease scoring of Stage 2 [17]. Additionally, R&K proposes two separate stages for SWS.

**Fig 1:**
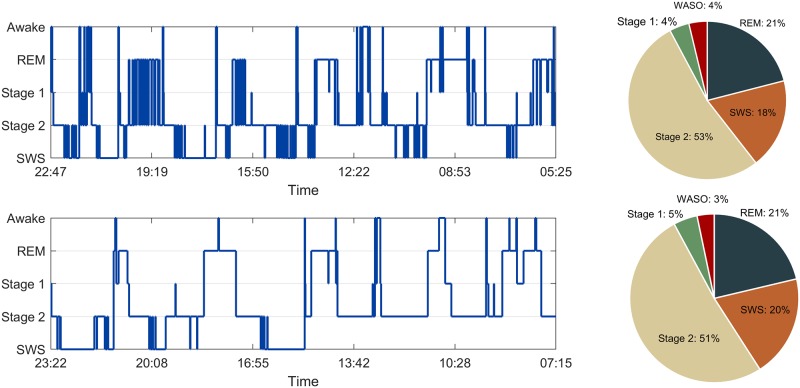
Hypnogram and corresponding stage proportions of fragmented sleep (top) and normal sleep (bottom). Stage proportions are the minutes in each stage normalized by total sleep time. REM: rapid eye movement sleep; SWS: slow wave sleep; WASO: wake after sleep onset.

A common clinical and research practice is to average the time spent in each sleep stage across a sleep period, and then normalize them by the total sleep time in order to quantify sleep stage proportions. While this approach is useful for group level differences, it reduces important variability in the pattern of stage transitions, and the durations of each sleep stage are lost[18]. This issue is highlighted in Fig 1 in which two qualitatively different hypnograms are shown—the first exhibits very fragmented sleep, with shorter bout durations for each stage and a higher number of stage transitions compared to the second. Nonetheless, when using stage proportions as a measure, there is no quantitative difference. Thus, considering that sleep fragmentation is a marker of detrimental, but treatable, health disorders such as obstructive sleep apnea[19], alternative measures should be considered.

Further, due to homeostatic and circadian processes[20], the distribution of sleep stages across the night is not uniform, and averaging stage proportions over an entire night of sleep loses potentially important information. Third, sleep proportion statistics collapse across duration and transition information, dropping valuable information about sleep fragmentation in the process. For example, if the proportion of the night spent in SWS decreased towards morning, sleep proportion statistics are not able to distinguish whether this is because the length of SWS bouts decreased (fragmented SWS) or if the subject simply transitioned to SWS less (while bout durations remained constant). If different mechanisms are involved in the transitions between stages and the maintenance of stages, then detection of their differing contributions is useful, but impossible with sleep proportion statistics. As we and others[18,21–25] have pointed out, sleep architecture is a time-varying signal, and temporally appropriate measures should be considered. To address this concern, we utilized two proposed dynamic measures: stage transition probabilities (the probably of transitioning to a stage from some other stage) and stage duration distributions (the distribution of time spent in an individual bouts of a stage)[22–30]. Both measures define parameterizable probability distributions, a desirable property considering that the complex pattern of sleep stages can only be partiality determined from observable variables.

Basic models of sleep dynamics already exist, the most popular of which is the two-process model[31] of sleep regulation. The two-process model defines two orthogonal processes, namely Process S and Process C, that drive sleep/wake transitions. Process S, or homeostatic sleep pressure, is a putative signal that rises exponentially during time spent awake and diminishes during non-REM (NREM) sleep as a function of slow wave activity. Process C, the circadian rhythm, is a cyclic signal based on the approximate 24hr biological clock of humans. The interaction of these two processes predicts sleep/wake dynamics. A third cyclic “ultradian” process has been added to the model[20] which deterministically (based on time since sleep onset) controls transitions between non-REM and REM sleep. While the two-process model captures global trends, it is a relatively simple model (i.e. does not account for Stage 1, 2 and SWS separately) and cannot account for many of the individual differences seen in real hypnograms. Moreover, the sleep architecture pattern of real sleep is far from deterministic and often exhibits alternate patterns. Some of these deviations from typical patterns may be indicative of underlying mental or sleep disorders and deserve closer attention while others may be more benign. A model that captures the probabilistic, temporal pattern of all stages (Stage 1, Stage 2, SWS, REM and WASO), accounts for individual variability seen in normal sleep architecture (or abnormal sleep, if trained on abnormal data), and generalizes to new, unseen data, would have scientific, clinical and translational relevance.

Here, we utilized the power of big data (~3200 nights of sleep) from 14 databases, to quantify the expected changes in sleep due to benign factors in the broader population. We began with a regression framework to investigate the relationship between age, sex and traditional, static sleep measures of stage proportions, sleep latency and sleep efficiency. We then moved onto a combination of Bayesian networks and temporally appropriate sleep architecture measures to examine sleep pattern dynamics. It is little understood how the dynamic patterns of sleep are influenced by individual factors. To investigate, we first begin with model selection algorithms to determine which variables must be considered when modeling sleep architecture dynamics, and which can be safely excluded to reduce model complexity. Next, we use the best fitting models determine how these variables modulate sleep dynamics. Subtle differences exist in the data collection methods of the 14 datasets analyzed in this study (e.g. different recording devices and alternate sleep scoring procedures). To minimize the effect of these nuisance factors, data analysis takes a multi-level approach whereby both the variance within and between datasets is quantified.

## Results

### Traditional sleep statistics

**Fig 2:**
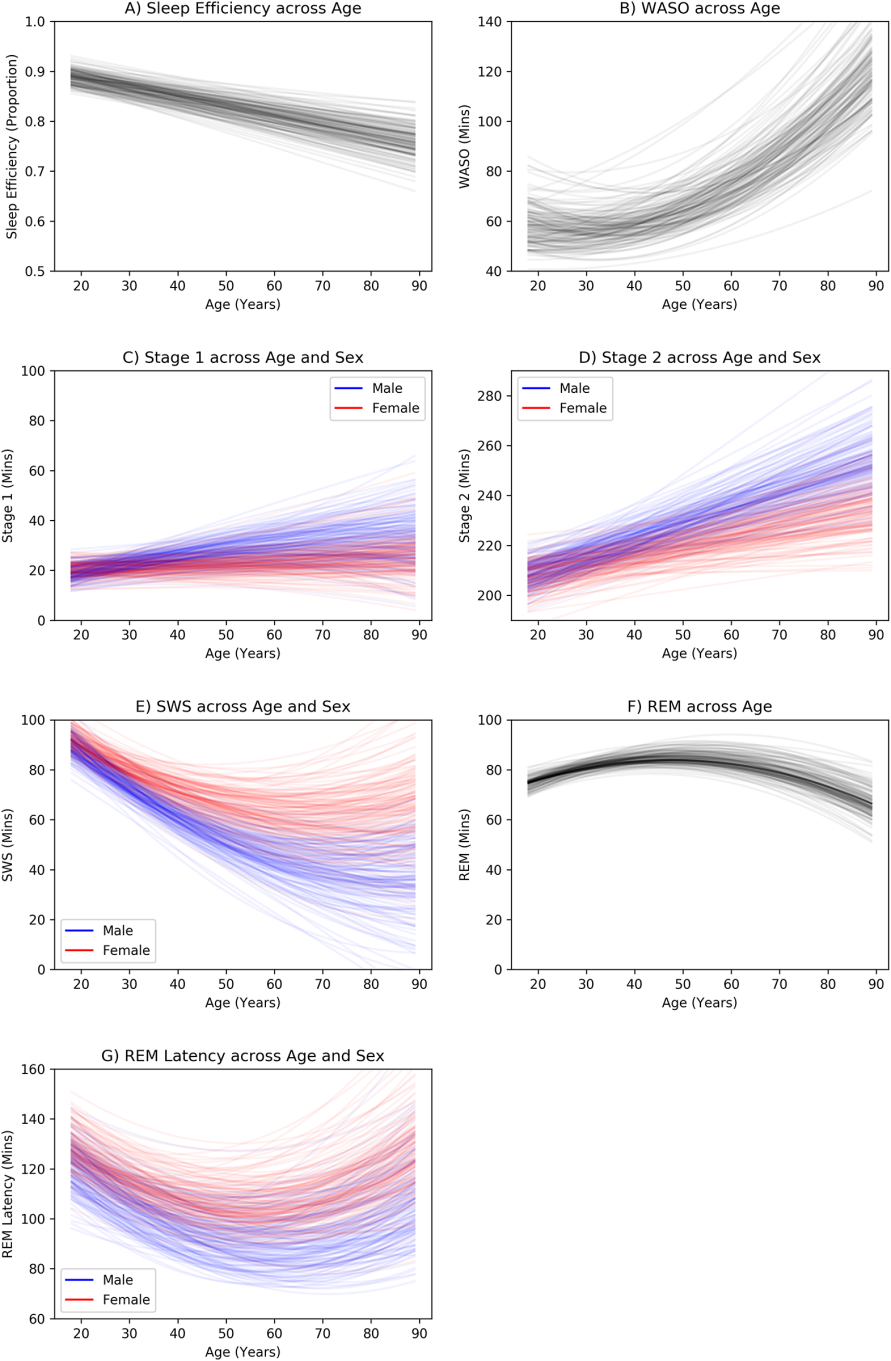
Static sleep statistics across age and sex. Top row: Sleep Efficiency, Sleep Latency, Bottom row: minutes in each stage. To show the uncertainly in predictions, regression parameters are randomly sampled 100 times from each model’s joint parameter distribution and each is used to plot a regression line. REM: rapid eye movement sleep; SWS: slow wave sleep; WASO: wake after sleep onset.

Using multi-level regression models, we predicted sleep efficiency, minutes in stage, and REM latency in a stepwise procedure from age, sex, and higher order terms while controlling for total sleep time (TST), and sleep onset time. While these analysis fail to capture dynamic trends, they remain useful as global summary statistics and for comparison with previous literature[6]. Regression parameters for the final best fitting models are reported in S1 Table, and each relationship is shown graphically in Fig 2 (where TST and sleep onset are fixed at their mean). For all relationships, a random slopes model better accounted for the data compared to a pooled or random intercepts model.

We began with sleep efficiency, which was best predicted with age, but not sex or any higher order terms (P(Correct Model | Data, Models) = 0.64, P(next best model, with age^2^) = 0.34). In general, sleep efficiency decreased with age at a linear rate of 2% per decade (B_age_ = -0.002, 95%CI = [-0.003, -0.001]).

We then modeled how the minutes in each stage changed as a function of sex and age. For WASO, a model with age and age^2^ variables, but not sex was selected (P(Correct Model | Data, Models) = 0.76; P(next best model, without age^2^) = 0.22). Here we find that WASO increases non-linearly across age, beginning at around 60 minutes at 18 years, remaining flat until 45 years, and then increasing sharply throughout middle and older age (B_age_ = -0.94, 95%CI = [-1.84, -0.19]; B_age2_ = 0.02, 95%CI = [0.01,0.02]). For stage 1, the model with age, sex, age*sex, age^2^ and the age^2^*sex interaction best explained the data (P(Correct Model | Data, Models) = 0.87; P(next best model, without age^2^*sex) = 0.08). For females, minutes in stage 1 increase only very slightly throughout their life (B_age_ = 0.06, CI = [-0.36, 0.50], B_age2_ = 0.000 [-0.004,0.004]). Males, on the other hand, increase by over half a minute for each year of life (B_age_ = 0.37, 95%CI = [-0.13,0.89]), however, this increase slowed somewhat with age (-0.002, 95%CI = [-0.008,0.003]). Stage 2 was best predicted from age, sex and the age*sex interaction (P(Correct Model | Data, Models) = 0.85; P(next best model, inc. age^2^) = 0.07). Minutes in stage 2 begin around 210 minutes for both sexes, and increase 39 seconds per year for females (B_age_ = 0.39, 95%CI = [0.09,0.76]), and faster, at over 1 minute per year for males (B_age*sex_ = 0.26, [-0.05, 0.57]). For SWS, age, age^2^, sex and the sex*age interaction were selected in the best model (P(Correct Model | Data, Models) = 0.86; P(next best model, inc. sex*age^2^) = 0.14). Both sexes spend less time in SWS as they age (B_age_ = -1.67, 95%CI = [-2.36, -0.91]), but this effect reduces and begins flattening off at ~70 years (B_age_ = 0.01, [0.00,0.02]). Males have less SWS than females, and have a sharper reduction of SWS with age than females (B_age*sex_ = -0.34, 95%CI = [-0.66, -0.07]). The best model for REM included age and age^2^ as predictors. This relationship was interesting, in that REM minutes begin low, and increased through to mid age, and then began to decrease again (B_age_ = 1.10, 95%CI = [0.76,1.53], B_age2_ = -0.01 [-0.02, -0.01]).

Finally, we investigated REM latency as a function of the same predictors as above. The full random slopes model, including age, sex, age^2^, age*sex gave the best fit (P(Correct Model | Data, Models) = 0.90; P(next best model, inc. sex*age^2^) = 0.09). Large main effects of sex are present, with females taking 7 minutes longer to enter REM than males (B_sex_ = -7.04, 95%CI = [-25.33,9.91]). REM latency reduces with age (B_age_ = -2.02, 95%CI = [-2.66, -1.30]), and more so for males (B_age*sex_ = -0.14, 95%CI = [-0.59, 0.25], however, the non-linear relation with age shows that younger and older adults take longer to enter REM than mid age adults (B_age2_ = 0.02, 95%CI = [0.01,0.03]).

Taken together, these results suggest that as men age, they spend more time in Stage 1 and 2, and this is offset with less time in SWS. For females, the same pattern exists, but changes across age are much less pronounced. For both sexes, WASO minutes remain constant in early life, but then begin increasing at around 45 years of age. REM minutes showed no changes across sex but a curvilinear relationship was observed where mid age adults have the most REM. Similarly, mid age adults are quicker to enter REM. Sleep efficiency declined linearly over the lifetime, driven, in part, by a rise in WASO.

### The dynamic pattern of sleep architecture

**Fig 3:**
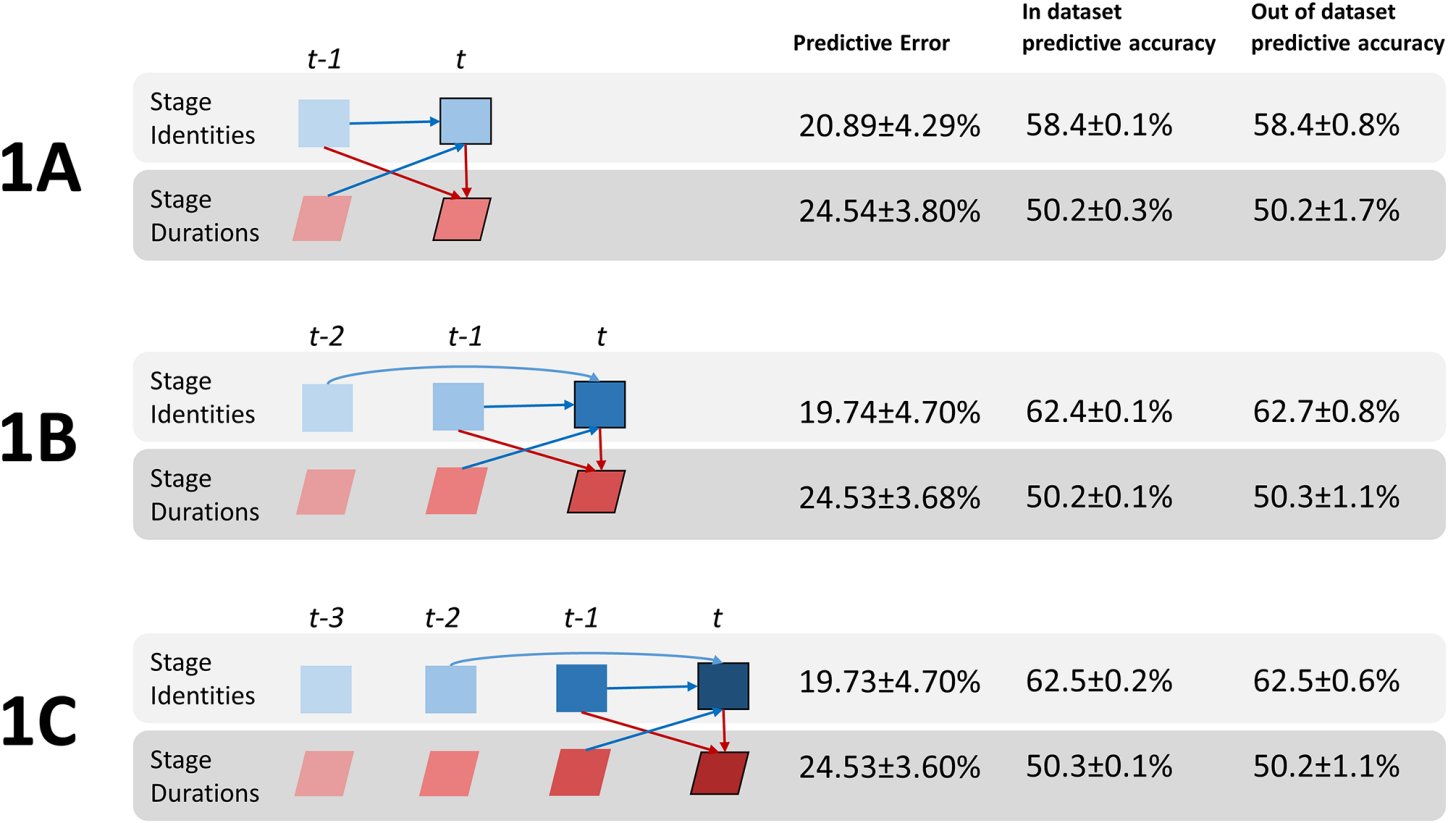
Best fitting models to predict the current stage and duration from previous sleep architecture variables. “In dataset” and “out of dataset” prediction accuracy and prediction error for current stage (top) and current stage duration (bottom) is shown. **Model 1A**) 1 back model (including t-1 variables), **Model 1B**) 2 back model (including t-2 variables), **Model 1C**) 3 back (including t-3 variables). When considering previous sleep archetecture only, Model 1B gave the best fit and states that the identity of the current stage is dependent on the identity of the previous 2 stages and the duration of the last stage (blue arrows). The duration of the current stage is dependent on the identity of the current stage (at *t*) and the previous one (at *t-1*) (red arrows).

Next, we turned to analysis using Bayesian networks to investigate the dynamics of sleep. The pattern of sleep stages over time is semi-deterministic. That is, the likelihood of the current stage and its duration is a probabilistic function of the stages that came before it, their durations, as well as factors such as time of day. It is clear from past literature[32] that some transitions are more or less likely (e.g. SWS->REM is unlikely, while SWS->Stage 2 is likely), therefore the identity of the previous sleep stage influences the current stage. We sought to determine the temporal structure of sleep architecture by testing the influence of previous stages (both identity and duration) on the current stage. For this purpose, we used the K2 structural search algorithm to find the best-fitting dynamic Bayesian network over sleep architecture variables. Three models were tested with varying amounts of previous stage information: one back (*t-1*), two back (*t-2*) and 3 back (*t-3*) (Fig 3).

Comparing Fig 3, Models 1B and 1C, we saw the identity of the current stage (at *t*), is optimally predicted by the two stages before it and the previous stage duration (at *t-1*). The duration of the current stage (at *t*) was probabilistic function of the identity of the current stage (at *t)* and previous stage (*t-1)*. The best model (1B) predicts the next stage at 62.5% accuracy and the duration of the next stage at 50.3% accuracy. Out of dataset accuracy is no different than in dataset accuracy, suggesting high model generalizability. Adding stage information from 3 stages back (i.e. t-3, Model 1C) does not aid predictive power (no connections from t-3 to t). Hence, the probability of the current stage may be modeled as a 2^nd^ order Markov Process and its duration a first order Markov Process. This is a promising result for future predictive models as it mitigates the computational explosion resulting from higher order processes. Having concluded that the stages and durations more than 3-back were independent of the current stage and its duration (given all other variables), we omitted them from future modeling.

Given the uneven age distribution present in the data (see *Dataset* section), there is a chance the model learns to predict sleep in older adults very well, and other age groups poorly, which averaged together leads to the reported performance metrics. To rule this out, we use the model to predict the current stage and its duration for each age group separately. Current stage accuracy was 62.4%, 61.6% and 62.8% and duration was 50.2%, 50.5% and 50.3% for the younger, mid age and older groups respectively. Given the small differences in these accuracies, we conclude the model has not overfit to the older age group.

### The influence of Time of Day and Time Slept

**Fig 4:**
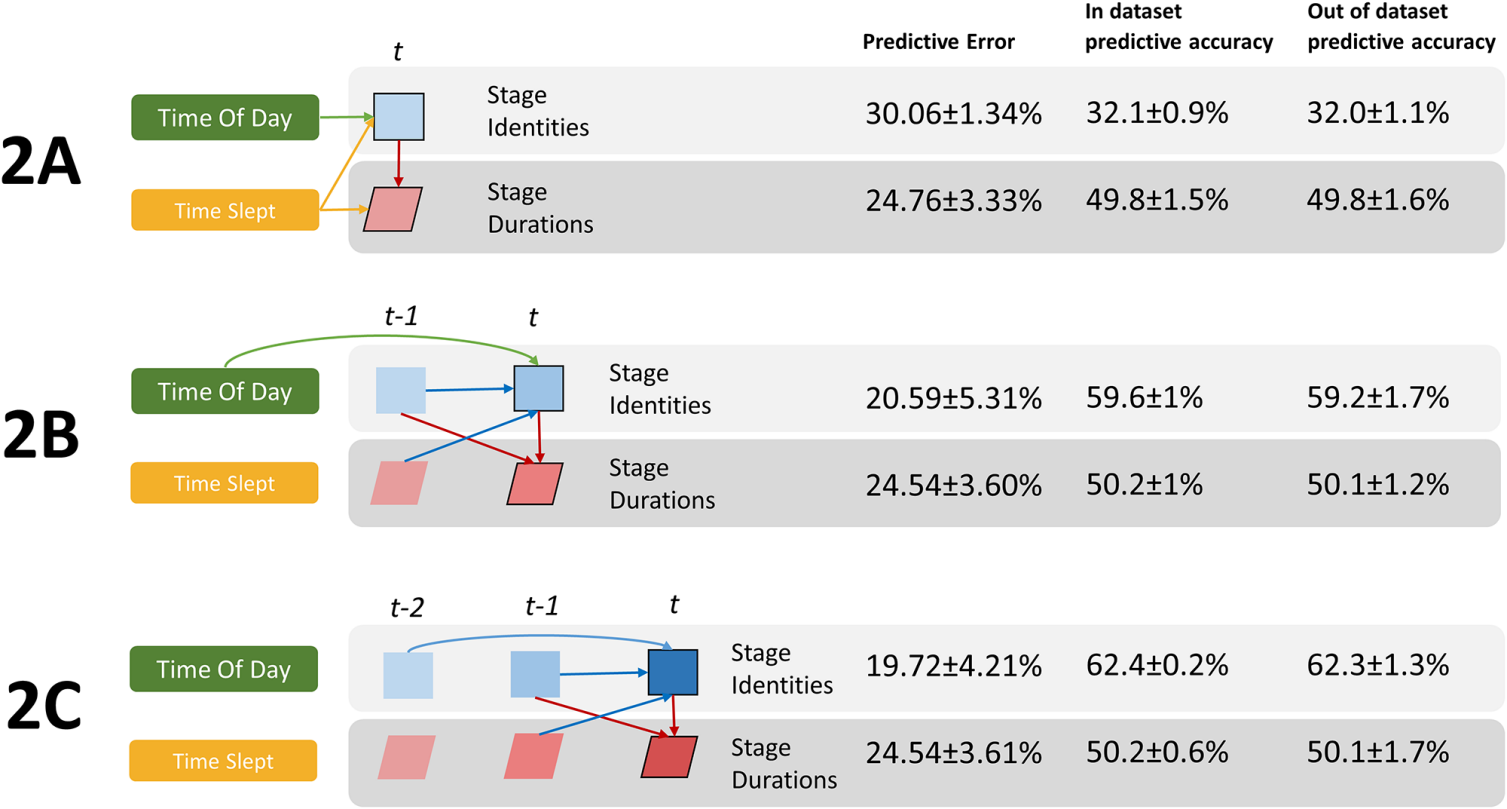
Effects of Time of Day and Total Sleep Time. **Model 2A**: When previous stages not included, **Model 2B**: 1 back included, **Model 2C**: 2 back included. Beside each model is the “in dataset” and “out of dataset” prediction accuracy and prediction error for current stage (top) and current stage duration (bottom). Time of Day influences 0^th^ order transition probabilities (2A) and 1^st^ order transition probabilities (2B). Total Sleep Time influences both 0^th^ order transition probabilities and duration distributions when no previous stage information is available (2A).

We added the *Time of Day* and *Time Slept* variables to the model to test their influence on the current stage and its duration. Three models were run: without previous stage information, with 1 back stage information, and with 2 back stage information (Fig 4, Models 2A, 2B and 2C).

We find that *Time of Day* affected the likelihood of the current stage when no previous stage information is available (i.e. 0^th^ order transition probabilities, Fig 4, Model 2A) and the probability of the current stage given the last (i.e. 1^st^ order transition probabilities, Fig 4, Model 2B). *Time of Day* did not aid prediction of the current stage when at least the last two stages were known (Fig 4, Model 2C). *Time Slept* influenced both the current stage and its duration, but only when no previous stage information was available (Fig 4, Model 2A). These findings are not surprising; the two-process model, and experimental observation[14], have shown variation in sleep architecture based on circadian timing (*Time Of Day*) and magnitude of sleep pressure (*Time Slept*). In addition to the two-process model, our model suggests that stage duration changes are influenced by the duration of time already spent sleeping, rather than circadian effects.

**Fig 5:**
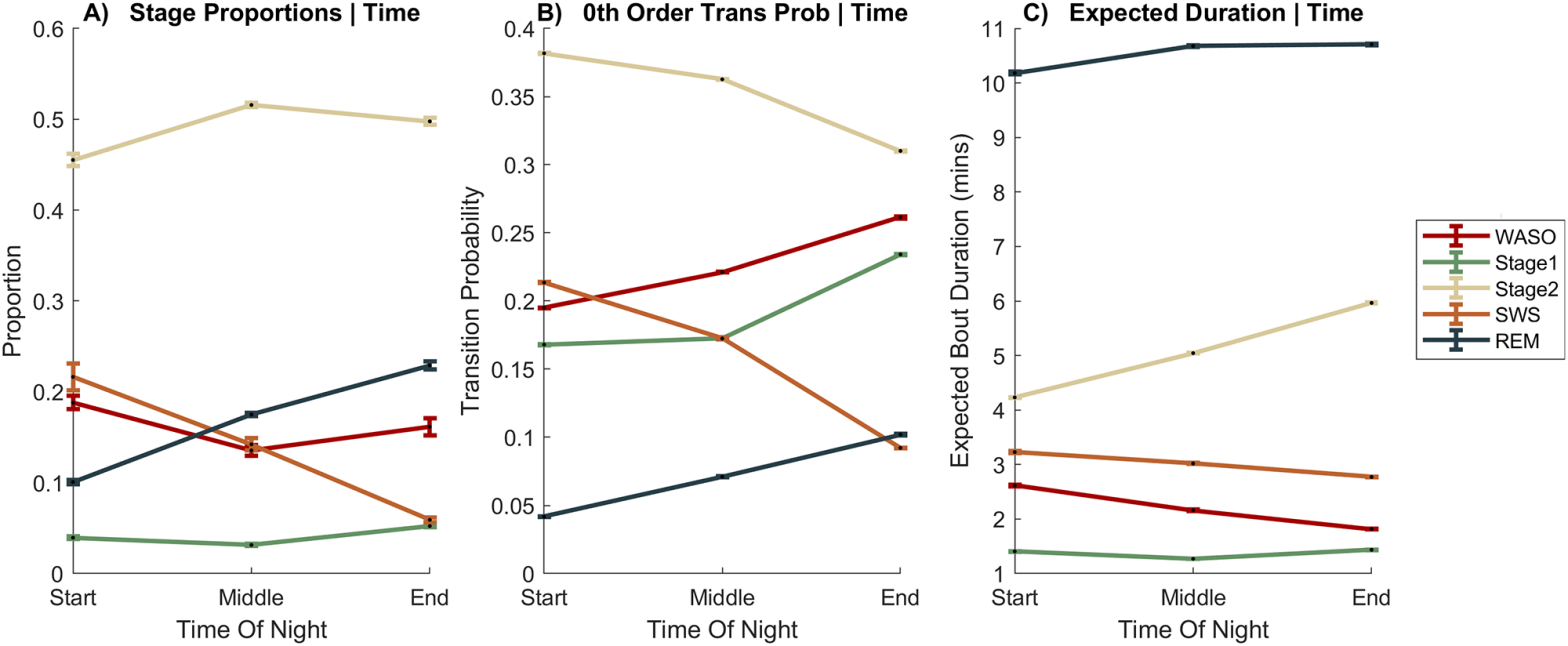
Effects on model parameters (Model 2A) across the night. **A)** Stage proportions, **B)** Transition Probabilities (0^th^ Order—the probability of transitioning to a particular stage from any stage), **C)** Stage duration distributions as measured by expected duration. To calculate each statistic, we ran the model 14 times, each time removing (and then replacing) one of the datasets from the full set of 14 datasets used to train the model. Points are the mean and error bars are the standard deviation across these 14 runs (see Methods). REM: rapid eye movement sleep; SWS: slow wave sleep; WASO: wake after sleep onset.

As sleep progresses through the night, there is a trade-off between the proportion of NREM and REM sleep. Prior work using stage proportions was not able to determine whether these temporal changes are due to an increased propensity to transition into NREM vs. REM or longer durations of NREM/REM bouts. To answer this question, we used our model (2A) to calculate the 0^th^ order transition probabilities (transition probabilities *from any stage*) and the expected duration for each stage at 3 points over the night: start (when *Time of Day* = 1, *Time Slept* = 1, 26% of data), middle (*Time of Day* = 2, *Time Slept* = 2, 19% of data) and end (*Time of Day* = 3, *Time Slept* = 3, 26% of data). We then compare these values with the traditional measure of stage proportions in Fig 5.

Stage proportions were as expected, with decreasing SWS and increasing REM from night to morning. Model parameters showed that the decrease in SWS proportion was driven by both a reduced tendency to transfer to this stage (Fig 4, Model 2B), and reduced bout duration (Fig 4, Model 2C), with the former decreasing sharply towards morning. The REM pattern across the night mirrored SWS, but overall REM had longer durations, and lower transition probability than SWS. Another interesting finding was that while WASO bouts became progressively shorter as the night progressed, they also became more frequent. It is often reported that the proportion of Stage 2 is constant across the night. However, our analysis showed Stage 2 transition probabilities decreased, but the durations increased over the night (less fragmentation towards morning), the effects of which, combined, left relatively flat stage proportions. Finally, we noted that model predictions do not differ substantially across age groups (< 0.5% variation for current stage, < 0.7% variation for duration).

### Adding individual factors

**Fig 6:**
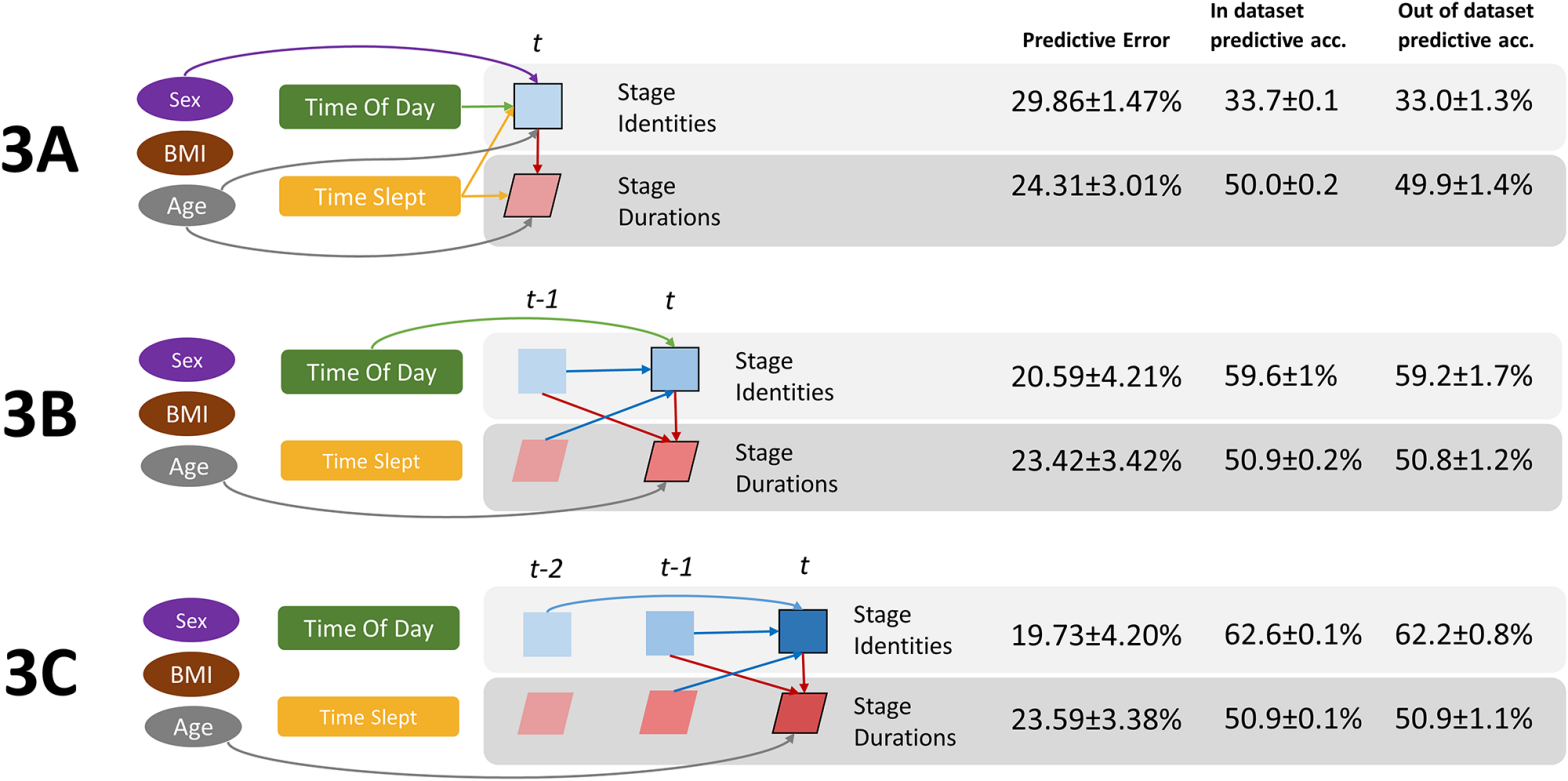
Full model including individual factors. **Model 3A**: No previous stage information, **Model 3B**: 1 back stage information, **Model 3C**: 2 back stage information. Beside each model is the “in dataset” and “out of dataset” prediction accuracy and prediction error for current stage (top) and current stage duration (bottom). BMI: Body Mass Index.

We added individual variables of *Sex*, *Age* and *BMI* to the models from above (Fig 6). We found BMI had no influence on stage durations or transition probabilities (when *Time of Day* and *Time Slept*, *Sex* and *Age* were accounted for). *Sex* modulated the probability of the current stage’s identity (i.e. different 0^th^ order transition probabilities for each sex group). *Age*, modeled categorically as younger (18–42 years), middle age (43–66 years), and older (67–90 years) groups, had profound effects on sleep architecture dynamics, influencing 0^th^ order transition properties (Model 3A), and influenced stage durations even when all previous stages that were predictive were included (Fig 6, Models 3A, 3B, 3C).

**Fig 7:**
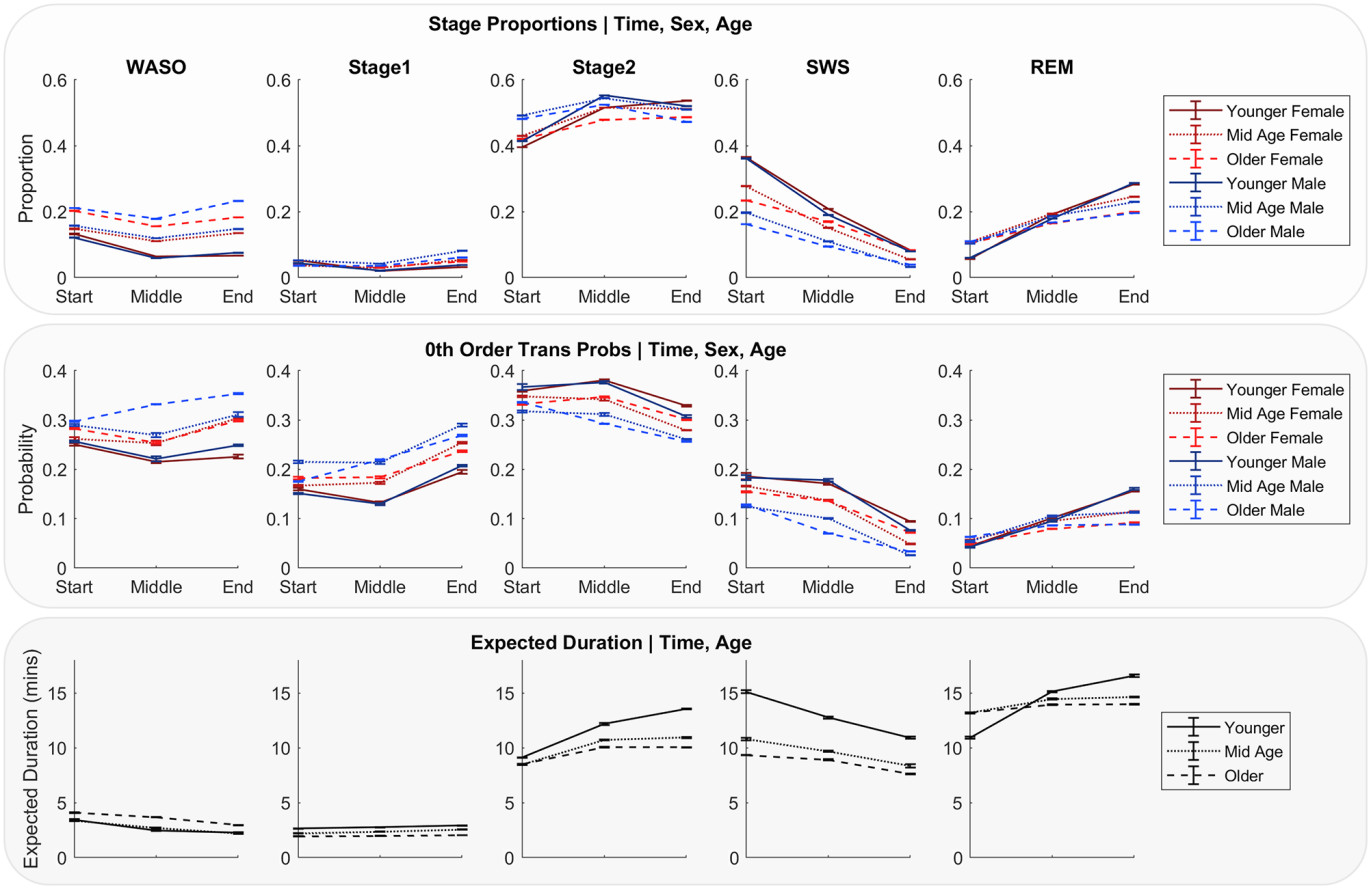
Model parameters, for each stage, age and sex group separately, as Time of Day and Total Sleep Time are increased across the night. Expected durations do not depend on sex, and therefore are the same for both sex groups. To calculate each statistic, we ran the model 14 times, each time removing (and then replacing) one of the datasets from the full set of 14 datasets used to train the model. Points are the mean and error bars are the standard deviation across these 14 runs (see Methods). REM: rapid eye movement sleep; SWS: slow wave sleep; WASO: wake after sleep onset.

The effects of sex and age on stage proportions, transition probabilities, and expected duration were captured in the parameters of Model 3A (Fig 6, Model 3A, Model 3B, Model 3C; Plotted in Fig 7). In general, durations were shorter and underwent less change across the night for mid age and older adults compared to younger adults (i.e. main effect of age, and an age*time interaction). Analysis of the duration distribution parameters (Fig 6, Model 3**C**) showed that REM duration was up by 50% from night to morning for younger adults, while REM duration in mid age and older adults only increased approximately 6%. Similar but flipped patterns existed for Stage 2 and SWS. In older and mid age adults, durations of NREM (SWS, Stage 2, Stage 1) were shorter, indicating more fragmentations. For Stage 2, SWS and REM middle age and older adult durations tend to cluster and are dissimilar to younger adults. However, for WASO older adults are clear outliers from their younger counterparts, experiencing, on average an extra minute awake for every time they wake up.

Transition probabilities for all stages were affected by age (Fig 6, **Model 3B**) and an interaction between age and sex was apparent. By middle age, transitions to deeper NREM stages (Stage 2, SWS) are less frequent, and this relationship is stronger for males. The reduced transitions to SWS and Stage 2 were counteracted by greater propensity to transition to WASO and Stage 1, a pattern that, again, begins at mid age and is stronger for males. Transitions to WASO are particularly strong for older males. REM differences with age are apparent at the end of the night, where mid age and older adults enter REM less than younger adults (main effect of age, negligible sex interaction, stronger for oldest group).

### Model prediction

While the purpose of our model is not a sleep stage classifier, it can be used to predict the probability of the next stage based on current information. The best fitting model overall was Model 3C (predictive error at 19.73% and 23.39%). When we used this model to predict the current stage information, accuracy was at 62.6% for stage identity and 50.9% for stage duration (chance is 20% for stage identify and 25% for duration). Note that regardless of model quality, the current stage is not 100% predictable given the variables considered. Testing model prediction accuracy for younger, mid age and older groups separately gives 62.4%, 61.9%, 63.2% for current stage, and 50.9%, 51.2%, 49.8% for duration.

Most of the predictive accuracy for the current stage comes from the stage directly before it (27.5%, Model 2A vs 2B), with only 1.2% more accuracy when including Time of Day in the model when 1 previous stage is known (Model 1B vs 2A). Similarly, age and sex produce a small increase of 1.6% on transition probability accuracy from the model without them (Model 3A vs 2A). For the current stages duration, accuracy does not improve markedly with the addition of previous stage identity (0.4%, Model 1A vs 1B), or age (0.7%, Model 2C vs 3C).

Bayesian networks are generative models, and the probability of predictor (parent) variable, such as age or sex, is easily computed from the outcome sleep architecture variables. Using this property, we trained the model (Model 3A) on all but 100 “hold-out” subjects, and then predicted the age group category (younger, mid age or older) of the untrained subjects. Each sleep data point was added to the model, and the most common age category returned over all data points was considered the age prediction. This was repeated 3 times, with a different set of hold-out subjects each time. The model was able to predict the correct age category 68±5% of the time (chance is ~33%).

## Discussion

Using a large multi-source dataset consisting of 3202 overnight recordings from a broad group of healthy people across a wide age range, we investigated sleep architecture and how it is affected by individual differences. First, we used static sleep statistics to show that sleep differed across both age and sex while controlling for total sleep time and sleep start time. Results match pervious meta-analysis work [6], where minutes in WASO, Stage 1, Stage 2 increased across age, while Slow Wave Sleep (SWS) and sleep efficiency decrease. For all NREM stages, we found significant sex*age interaction, where males traded off an increase in lighter NREM minutes (stage 1, stage 2) for a decrease in SWS minutes. In contrast to previous work [6], REM minutes, and REM latency both showed curvilinear relationships where mid age adults had decreased REM and reduced time to first enter this stage. These REM findings may point to sleep deprivation in this group, where early work schedules restrict morning REM, which in turn causes early transitions to this stage in subsequent nights.

Using discrete Bayesian networks, we then quantified the patterns and durations of sleep stages, and potential moderators of this process. In keeping with the two-process model, we found influence of Time of Day (affected transition probabilities only) and Time Slept (affected both transition probabilities and stage durations) on sleep architecture. Interestingly, we showed that relatively flat profile of WASO and Stage 2 usually observed across the night was a combination of rising transition probabilities and falling durations for WASO, and falling transition probabilities and rising durations for Stage 2.

When adding individual differences, we found BMI had no effect, and that sex affects transition probabilities only. Sex’s influence on transition probabilities, as opposed to durations, suggests that sex differences in sleep architecture are not due to a reduced tendency to stay in various stages, but difficulty in transitioning to them. Age modulates transition probabilities and durations, even when all previous stage information is available and, similar to a previous investigation of transition probabilities[28], we find older adults have a reduced tendency to transition to SWS, REM and wake more during the night. Considering each age/sex group separately, we found that middle age and older adults, and males in particular, had a reduced tendency to transition to SWS and shortened SWS durations. A reduction in SWS proportion for older males compared to older females has been reported elsewhere[6]. Here, we showed that this is due to a reduction in transition to SWS as opposed to shorter SWS durations. Mid age and older adults had more fragmented sleep, exhibited by reduced duration of SWS and morning REM. This finding may suggest a deterioration in the neural mechanism that sustain SWS across age.

The current results do support the idea that sleep architectural patterns are semi-deterministic—that the current stage’s probability is driven by stages that occurred before it. Even without considering specific features of PSG, our model could predict the next stage to be transitioned to, and its duration, approximately 62.6% and 50.9% of the time respectively. Much of this predictive power was gained by previous sleep architecture information alone, and not individual differences or time of day/time slept. We conclude that future automatic sleep scoring algorithms should include temporal context (i.e. previous stage information) to maximize detection accuracy, but temporal context alone is not enough to provide robust scoring. While age did not add much predictive power to the model, the model was able to predict the age category from sleep architecture (68±5% accuracy). Future models may have success taking a similar approach to detect illness from a combination of sleep dynamics and other indicators of pathology.

In Markov Chain models of sleep architecture[18,27,33], the transition to the current stage is considered a random process and is dependent on only the previous stage (*t-1*), or no stages at all. Our work concludes that sleep is a 2^nd^ order Markov Process and the addition of the stage 2 back (*t-2*) is important. Further, these data highlight that if predicting the current stage is the only goal of a model, and previous stage information is given, then *Time of Day/Time Slept* information may be irrelevant (but age information remains important). This is likely because specific patterns of stages (and their durations) occur at different times over the hours of sleep, and once a specific pattern has initiated, then *Time of Day* provides no extra information to predict the pattern end.

Contrary to previous literature, we found no relationship between BMI and sleep architecture. Rao *et al*.[34] reported increased BMI associated with decreased proportion of SWS in an older population (MrOS Study) after controlling for age, sex, clinic location, race, sleep efficiency, sleep disordered breathing and other health variables. Additionally, Redline et al.[8] found increased lighter sleep stages and reduced SWS associated with low BMI. These studies had less stringent BMI exclusion criteria, looked at stage proportions rather than duration distributions and transition probabilities, and summed SWS over the whole night. These differences may account for our alternative BMI findings. BMI information was missing from some studies, and the lower number of data points may have contributed to the lack of a BMI finding. However, after removing data without BMI information and running all models without BMI, the best fitting relationship among variables was unchanged for all, suggesting that a lack of statistical power was not an issue. Further, while we employed strict exclusion criteria to remove sleep disorders, some undiagnosed or subthreshold illnesses may be present. It remains plausible that the pooling of undetected disorders with normal sleepers may have blurred potential BMI relationships.

Similar to previous work[6,35,36], the current study found large effects of age, particularly on SWS and WASO. Our Bayesian network and regression analysis both found non-linear age effects with these stages whereby SWS minutes, proportions, durations and transition probabilities reduced sharply between youth and mid-age, but flattened off in later life. For WASO, the opposite was true, this stage was more constant up until mid-age, and then increased abruptly (for both minutes + durations). Existing literature provides several potential explanations for increased WASO with older age, including increased need to urinate during the night (nocturia)[37], increased anxiety, discomfort or pain from chronic illnesses and change in hormones (such as melatonin)[38]. In particular, previous reports indicating reduced melatonin levels[39,40] (a neurohormone related to circadian regulation and initiation of sleep), which decreases with age, may be one mechanism driving older adults increased tendency to transition to WASO. Increased presence of b-amyloid and neurofibrillary tangles in the aging brain[41], which has recently be linked to sleep fragmentation[42], as well as cell and receptor loss in of brain areas responsible for maintaining sleep homeostasis[36], may also account for the observed disrupted sleep with age.

Interactions between sex and age were apparent, particularly in both static and dynamic measure of SWS, and dynamic measures of WASO. Males had greater deficits in SWS as they age, exhibited by less total minutes in this stage, and reduced proportions, transition probabilities and durations. The sex hormone testosterone plays an import part in healthy, consolidated sleep[36] where reduced levels of testosterone lead to reductions in SWS[43]. Older males exhibit reduced levels of testosterone, and this may contribute to shorter durations, increased fragmentation of SWS and increased transitions to wake for the older male group. Furthermore, in some females, sleep disruption increases after menopause[44]. Postmenopausal women also exhibit increased SWS[45], which may account for the slight increase in SWS minutes in older females observed in static measures of SWS. Future studies should classify females into pre/post menopause, and also consider the hormonal cycle, which has recently been shown to impact REM architecture[46] and quality of NREM sleep[47].

### Limitations

Several limitations of the current study need to be acknowledged. First, there are limitations introduced by the data used. The data used has multiple levels of hierarchical, nested structure where subjects are nested in dataset, and many sleep architecture data points within a subject share that subject’s age, sex and BMI. While the nesting of data points within a dataset was considered, the nested structure of data points within a subject was ignored, hence our parameter estimates may be less accurate. Additionally, by using discrete Bayesian networks, the resolution of many naturally continuous variables (stage durations) is lost. However, the loss of power to detect variable relationships that stems from discretization[48] is, in part, mitigated by the large sample size used. To reduce predictive error and improve parameter estimates, future modeling work will focus on a hierarchical continuous model of the same data/variables.

Sleep stages were quantized into 30 second epoch blocks (already present in the data), a length which is arbitrary and chosen purely for historical reasons. In reality, sleep stage transitions do not occur only at 30 second boundaries, and future work should consider sleep stages as continuous. Sleep staging data was smoothed such that REM, SWS and Stage 2 bouts broken by 1 minute or less of another stage were merged. This ‘theory driven’ as opposed to ‘data driven’ approach was employed so that modeling tracked the duration of the well understood ultradian cycles, and smoothed noise created by incorrect scoring. Additionally, it smoothed over briefly fragmented stages to create a bias towards longer stage bouts. To test if the amount of smoothing was appropriate, we reran the final model with twice as much smoothing. Model structure did not change, and the overall pattern of parameters remained unaffected, however, there was a predictable bias towards longer durations of Stage 2, SWS and REM as more gaps of Stage 1 and WASO are filled. We also noticed that the transition probabilities of WASO and Stage 1 trivially reduce as some bouts of these stages have been filled (and therefore cannot be transitioned too).

In general, the multi-level regression and Bayesian analyses agree with each other. However, our dynamic Bayesian analysis detected older males transition to WASO far more than their counterparts, while this age*sex interaction was not clear from static measures. The observed discrepancy may be due to differences in these measures (e.g. the way total sleep time, or time slept is accounted for), or differences in how these model compute parameters from the multiple datasets used to train them. Higher resolution continuous Bayesian models may shed light on the age*sex interaction of Wake After Sleep Onset.

Potential confounds exist in our data. While we excluded anyone taking sleeping medication and anti-depressants, as well as anyone with reported neurological, psychiatric or sleep disorders, other medications or illnesses may affect sleep (e.g. Beta Blockers[49], diabetes[50]) and subjective sleep complaints were not taken into account. It is likely that undiagnosed or subthreshold, mental, physical or sleep disorders are present in the data. These potentially unhealthy individuals likely suffer from a range of disorders with differing effects on sleep architecture, making it impossible to detect or control for. Furthermore, detailed information of previous sleep history and habits were not available, and napping, chronotypes and circadian alignment were not considered. Napping is likely to be more apparent in the older population and may have influenced our results. Sleep scored with R&K vs AASM standards have shown different sleep architecture profiles[51]. While there is not enough numeracy in the AASM data to directly compare these two scoring methods, we reran the final model (Fig 6) without AASM data (4 datasets removed) and the conclusions drawn from this model did not change. Another issue that may impact differences across age group is that the EEG features used to visually score sleep change with age[52]. Potentially, some of the observed differences in sleep in older and young adults may be due to differences in classification of sleep epochs in these populations. Lastly, *Time of Day* and *Total Sleep Time* are two correlated parameters, and this may have reduced our ability to tease apart their effects. Future models should consider the addition of data from naps or circadian misalignment studies to add greater *Time of Day* vs *Total Sleep Time* variability. Additionally, a continuous model with more resolution of these variables may provide insightful information.

### Future directions

The addition of EEG variables to the model would greatly improve prediction of sleep stages as the sleep homeostasis literature has shown Slow Wave Activity is highly predictive of the propensity for sleep (Process S in the two-process model) and is correlated with NREM/REM cycles. Other features of the EEG are also indicative of specific sleep patterns (sleep spindles, K-complexes, REM events, alpha activity). However, by not including EEG explicitly in the model, we have developed an algorithm that can predict and simulate sleep stages based on data that is incomplete (missing some previous stage information, i.e. model 3B, 3C, 3B, 2B) or sensor-less (no previous stage information, i.e. model 3A, 2A). Sensorless models may be of commercial interest in ‘smart alarm’ products aimed to wake a user in a specific sleep stage to overcome the negative effects of sleep inertia[53], to architect the perfect nap[54], or to reduce the risk for early morning cardiovascular events and stroke[55].

Ethnicity information was not available for all datasets, however, given the genetic influence on sleep[56,57], an investigation of the changes in sleep dynamics with ethnicity is a future direction.

Our model should be considered a promising initial step towards a full continuous model of sleep architecture, able to accurately predict and generate the pattern of sleep stages over the night. One potential extension of our work is the diagnosis of sleep and health disorders including, but not limited to, excessive drug use, cardiovascular disease, diabetes, depression, sleep apnea and insomnia[58]. This would be readily achieved using the current modeling framework with the addition of variables that code for the presence of an illness. After training the model on a relevant dataset, it would then be possible to query the model on individual cases and garner a probability for having the target illness (similar to how we predicted age from sleep architecture).

### Conclusion

Our work demonstrates the high fidelity of Bayesian networks coupled with big data in capturing the dynamics of normal sleep and the influence of individual differences. Our model successfully captured the statistics of sleep architecture to predict the current stage. It shows that time of day, total sleep time, sex, age, but not BMI influence sleep architecture when no previous stage information is known. Future work will use continuous Bayesian network modeling on clinical datasets to help diagnose illness.

## Materials and methods

### Dataset

The 3202 records of sleep (mean age 62.5 years, 60% male) used to train the models in this study came from the following 14 sources: The University of California, Riverside (now Irvine) Sleep and Cognition Lab[59], Furman Sleep Lab[60], The Institute of Medical Psychology and Behavioral Neurobiology at the University Tübingen[61–69], the Sleep Psychophysiology Lab at University of Padova[70], 6 open-source sleep datasets available on the National Sleep Research Resource (NSRR)[50,71–83], the Sleep EDF Database[84–86], CAP Study Database[84,86], St Vincent/University College Dublin Dataset and the Montreal Archive of Sleep Studies[87] (MASS). The University of California Riverside Institutional Review board determined no new Ethical Review was required as the original ethics approval of each dataset covered the reuse of data for the current analysis. For datasets containing multiple nights for the same subjects, only the first night was used. Detailed information on the datasets can be found in supplementary material (S2 Table). Total sleep time is restricted in some studies and controlled for in analyses. Wake before sleep onset is removed from all records. BMI information was not available in some studies (see S1 Table for details). Models where BMI is a predictor ignore subjects without BMI (23% of subjects excluded).

**Fig 8:**
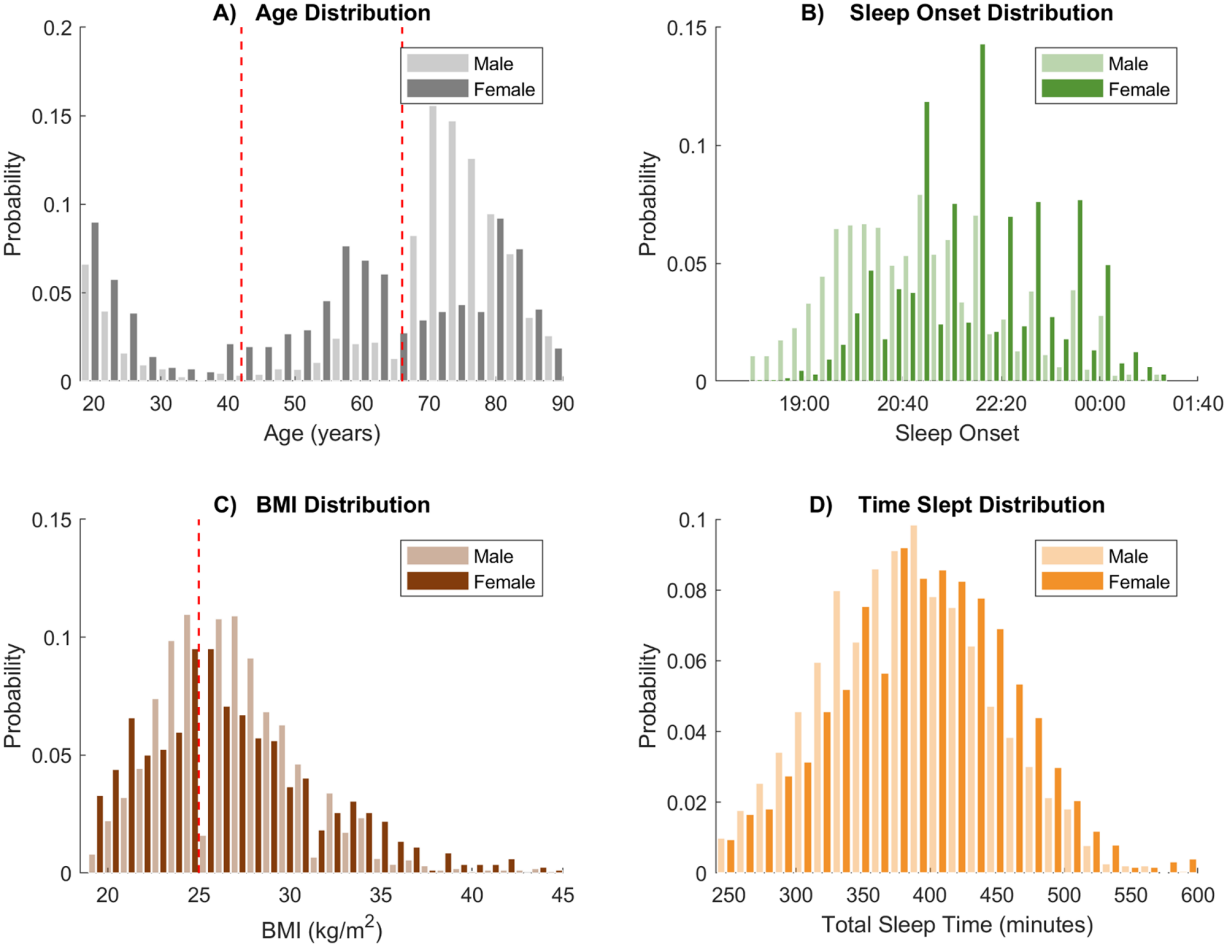
Distributions of continuous variables. Red dashed lines indicated edges of discretization where relevant (see *Discretization* section). A) Age (per subject), B) Time of Day (per data point), C) Body mass index (BMI, per subject), D) Time Slept (per data point—e.g. a subject who slept for 400 mins will impact the histogram from 0 to 400).

Records from individuals with diagnosed sleep disorders (e.g., narcolepsy, sleep apnea, sleep disorder breathing, and insomnia) and mental disorders/neurological disease (e.g., depression, schizophrenia, Parkinson’s, Alzheimer’s, etc.) were excluded. Subjects who reported taking anti-depressants or sleep aids were not included. Additionally, people that exhibited more than mild levels of sleep disordered breathing during the sleep recording were excluded (if apnea hypopnea Index > 15, obstructive apnea-hypopnea index > 15, obstructive apnea index > 5, central apnea index > 5, or respiratory disturbance index > 15). In datasets that contained subjects with known disorders affecting sleep, only control subjects were used (e.g. sleep-EDF Database). Records from people older than 90 years of age and younger than 18 years of age were excluded due to low numericity. People with extreme BMI values >50kg/m^2^, and those in the underweight category (<18.5kg/m^2^) were not considered. Subjects with extreme (±2.5 Standard deviations) sleep start times [<18:14, >01:20] and total sleep times [<285 mins, >650 mins] were considered outliers and removed. While these exclusion criteria sought to remove any unhealthy subjects, we cannot rule out the presence of some sub-threshold or undiagnosed mental or sleep disorders. Fig 8 shows distributions for continuous demographic variables for both sexes. Sleep records were scored using a combination of Rechtschaffen and Kales[15] (R&K) and the American Academy of Sleep Medicine[88] (AASM) standards (see S1 for each dataset) and Stage 3/Stage 4 Sleep from the R&K criteria were combined to give SWS. A comparison of PSG variables (Minutes in stages, Sleep Efficiency, Total Sleep Time) across dataset can be seen in S1 Fig.

### Static sleep architecture methods

We investigating the effect of individual differences on static measures of sleep architecture via a multi-level regression framework. Variables that sum to one are not generally suitable for linear regression techniques. Therefore, we excluded sleep proportions (the total time spent in a stage, normalized by total sleep time). Thus, we considered as dependent variables: minutes in stage, sleep efficiency (i.e., the ratio between total sleep time and the time spent in bed after sleep onset, proportion) and REM latency (i.e., the time from the first sleep stage to the first REM stage, minutes). Given the presence of sleep time restrictions in some studies and changes in sleep architecture across the day, we controlled for total sleep time (z-scored) and sleep onset (z-scored). Sex is coded 0 = female, 1 = male and age is left uncentered. Regressions are fit with a sample based Markov Chain Monte Carlo (MCMC) method (No U-Turn Sampler[89]) using the python package PyMC3[90]. The MCMC sampling procedures used 2 chains, a 500 sample burn in period and 2000 samples for parameter distribution estimates. Gelman-Ruben R[91] and visual inspection were used to monitor convergence.

To account for possible dataset differences (as seen in S1 Fig), we use a multi-level analysis and add variables in a stepwise manner, stopping once model fit does not improve. First, we began with a pooled model (non-multi-level) and test the simplest model, which includes the intercept term and covariates only (TST and sleep onset), then we add age, sex and finally the sex*age interaction, age^2^, and finally the sex*age^2^ interaction term. Second, we moved to a random intercept model (intercepts allowed to vary between datasets) and tested the same variables. Third, and finally, we progressed to random slopes model (slopes allowed to vary between datasets) with the same stepwise procedure. If including a variable increased model fit, then that variable is considered a significant predictor of the outcome. The 95% creditable intervals around parameter estimates are also reported. Models are compared using the Wannabe-Akaike Information Criterion (WAIC)[92], a generalization of the Akaike Information Criterion[93] (AIC), to measure out of sample fit expectation and control for overfitting. To select between candidate models, WIAC values are converted to Akaike weights, which give the probability that a particular model is the best model given the data and the set of candidate models[94].

To gain an intuitive understanding of relationships defined by the models, we plot predictions of outcome variables across age for male and female (Fig 2). To show the uncertainly in predictions, regression parameters are randomly sampled 150 times from the model’s joint parameter distribution (for each sex level), and each is used to plot a regression line.

### Dynamic sleep architecture methods

Bayesian network[95] methods proceeded in 3 steps: 1) we modeled the temporal structure of sleep by fitting a model to predict the current stage and its duration from previous stage information. 2) We tested the influence of other variables on sleep architecture by first introducing *Time of Day/Time Slept* to the model, and then 3) we tested the individual difference variables of sex, BMI and age. For 2 and 3, we varied the amount of previous stage information available to find the optimum predictive model. We sought to determine which candidate variables or set of candidate variables best predicted sleep architecture. Therefore, for all Bayesian models, a greedy search algorithm (K2 hill climbing algorithm[96]) was first used to find the best fitting model over the variables given (described in *Structural search and model comparison* section) followed by analysis of model parameters to elucidate the specific effects of age, sex, BMI, and time. Predictive error and accuracy was used to compare models (see *Structural search and model comparison*).

#### Dynamic measures: Transition probabilities and stage duration distributions

Stage transition probabilities have been used previously to detect sleep architecture changes with illness[23] or age[28] and are defined as the probabilities of transitioning to a specific stage from any stage i.e. P(Current Stage = i), to a specific stage from a specific stage, i.e. P(Current Stage = i|Previous Stage = j), or to a specific stage from some history of stages, i.e. P(Current Stage = i|Previous Stage = j, Previous Previous Stage = k) and are referred to as 0^th^ order, 1^st^ order, and 2^nd^ order transition probabilities respectively. For example, the 0^th^ order probability of REM, which is the probability of transitioning to REM from any stage would be low at the start of the night, and high towards the end of the night. However, the first order probability of transitioning from SWS to REM would be close to zero across the entire night. Probabilities were calculated by counting the number of transitions to stage *i* (or to stage *i* from stage *j*, etc.) and normalizing by the total number of stage transitions. First order transition probabilities can be seen in Table 1. We were invested in the dynamics of sleep once sleep has begun, rather than sleep/wake homeostasis, therefore transitions from initial wake or to final wake were not considered (i.e. WASO transitions includes spontaneous awakenings only).

**Table 1:** 1^st^ order transition probabilities static across whole night.

*1*^*st*^ *OrderTrans Probs*		TO STAGE:				
		WASO	Stage 1	Stage 2	SWS	REM
FROM STAGE:	WASO	-	0.64	0.33	0.00	0.02
	Stage 1	0.43	-	0.52	0.00	0.05
	Stage 2	0.44	0.04	-	0.31	0.21
	SWS	0.15	0.00	0.80	-	0.05
	REM	0.60	0.09	0.31	0.00	-

**Notes**: Data from all subjects and across whole night. REM: rapid eye movement sleep; SWS: slow wave sleep; WASO: wake after sleep onset.

#### Stage duration

**Fig 9:**
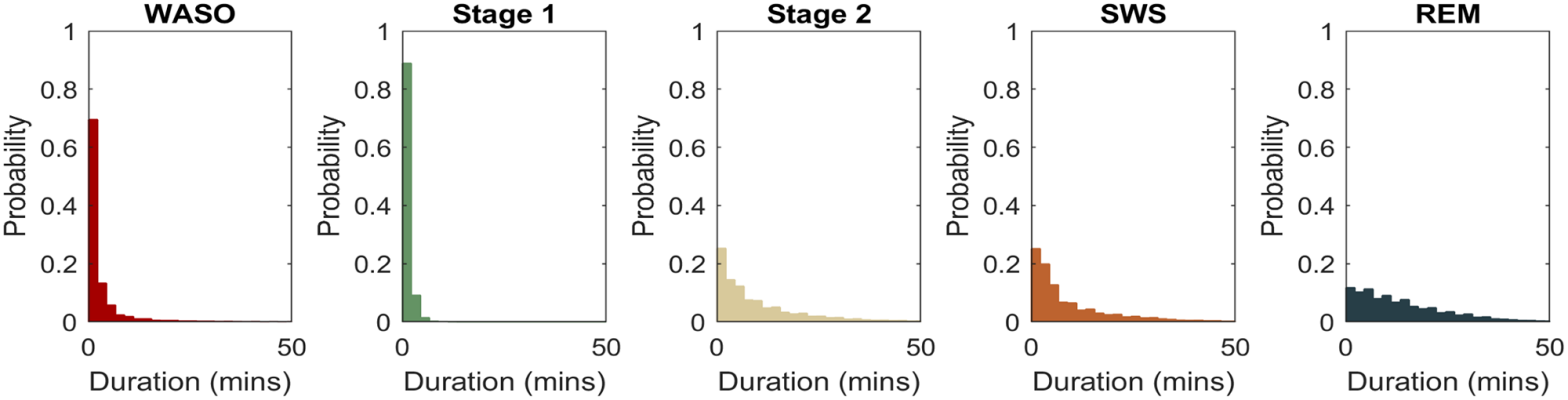
Stage duration distributions. Data from all subjects and across the whole night. REM duration distribution shows less fragmentation (longer bouts) than SWS. REM: rapid eye movement sleep; SWS: slow wave sleep; WASO: wake after sleep onset.

Distributions are the distributions of a specific stage’s bout durations. Here, bout duration refers to an uninterrupted segment of a specific stage. For example, one can extract all bouts of REM from a person or a group of people, and plot a histogram of their frequency with respect to duration (see Fig 9 for an example). If REM bouts are shorter (i.e. more fragmentation), then this histogram will have more probability mass at shorter durations. Sleep follows an ultradian pattern alternating between NREM and REM, and these ultradian periods, especially REM, may be broken by brief awakenings or transitions to Stage 1. To ensure that duration distributions account for ultradian period in their entirety, we smooth (in order) REM, SWS and Stage 2 such that gaps in these stages up to 1 minute in length are filled. Smoothing employs the “binary close” operation of dilation followed by erosion common in computer vision[97].

By calculating the transition probabilities and duration distribution histograms at different times of the day, it was possible to tease apart if a higher stage proportion statistic was due to increased likelihood to transition to that stage (e.g. more fragmentation) or extended durations of that stage (more rightward skew of duration distributions). Both of these measures were contained in the parameters of the Bayesian network when used to predict the current sleep stage. Note that transition probabilities and stage durations were not significantly correlated (e.g. in our data for WASO, r = 0.00, p = 0.99, n = 3202).

#### Variables considered

Before we can use dynamic measures to classify differences in sleep we needed to understand which variables modulate sleep architecture. Using discrete Bayesian networks, we tested the influence of various potential moderators (and, implicitly, their interactions) on sleep architecture. These models tested a series of independence assumptions. For example, we could ask if a model that defines a dependence between *time of day* and *stage transition probabilities* fits our data better than a model where these two variables are independent. Furthermore, we could test if the sleep architecture signal is quantitatively different if it came from younger or older adults, males or females, etc.

The variables tested are broadly categorized into individual differences (*BMI*, *Sex*, *Age*) and those theoretically derived from the two-process model (circadian cycle, modeled as *Time of Day*, and *Time Slept*). As opposed to Bedtime and Total Sleep Time which are a constant for each subject, *Time of Day* and *Time Slept* are updated across the night and capture the temporal state of a subject’s sleep. Note that *Time of Day* and *Time Slept* were correlated—they both increased over the night (r = 0.76, p<0.05)

We excluded the following variables: 1) EEG-based variables in the time and frequency domain (e.g., k-complexes, slow wave activity), which may have aided prediction and classification, but were left out of the current model to reduce complexity and allow the prediction of sleep with no physiological measurement (for models that include no previous sleep stage information); and 2) Prior sleep information (e.g., morning wake time), which would have provided additional information about homeostatic sleep pressure, but were not available in the current dataset.

### Bayesian networks

#### Structural search and model comparison

A Bayesian network is a probabilistic, generative, graphical model that represents a set of conditional dependences between random variables. The lack of a relationship between any variable A and any other variable B represents conditional independence between A and B. For example, if *sex* influences *sleep stage duration* and *sex* also influences *BMI*, then there will be a relation between *BMI* and *sleep stage duration* if *sex* is not included, but no relation if it is included in the model (i.e., BMI and stage duration are independent given sex). We used the Bayesian information criterion (BIC) score[98] to compare relationships between variables. This score captures the best fitting model while penalizing excess relations between variables to reduce overfitting (see S2 Fig in supplementary methods for an example). Bayesian networks were implemented using the Bayes Net Toolbox[99] for MATLAB[100].

The BIC score is only comparable across models with the same variables and data. To facilitate comparison across all models, we calculated each model’s *predictive error* and *predictive accuracy* of the correct current stage and duration from all other variables using a standard k-fold cross validation procedure (k = 5). This process ensured overfitting did not bias our results because no data used to train the model is used to estimate accuracies. The data was randomly shuffled and split into 5 equal folds. Four of these folds (80% of data) were combined to form a training set, and the remaining 20% of data was used for testing (testing accuracy reported only). The whole procedure was repeated 3 times with different random shuffles. The mean and standard deviation for all 5 splits and all 3 shuffles are reported for each model that appears in the paper.

*Predictive error* for each data point is the mean of the absolute difference between predicted stage probabilities and the actual stage observed (in vector form). For example, if the next stage is REM, the actual stage vector is [0, 0, 0, 0, 1] (here REM is the fifth stage). Our model may return the probability of the current stage as [0.2, 0.1, 0.1, 0.15, 0.35]. *Predictive error* for this data point is the mean of the absolute difference e.g. [0.2, 0.1, 0.1, 0.15, 0.65] = 0.24). Internally, the way each model attempts to maximize the BIC score and parameter estimates is equivalent to minimizing the *predictive error*. *Prediction accuracy* for each data point is the most probable stage (or duration bin) compared to the actual stage and takes a value of 1 if the most probable stage equals the actual stage, and zero otherwise. In the above example, the most probable stage is REM (P(REM) = 0.35) and the correct stage is also REM, therefore the predictive accuracy is 1 for that that data point. *Predictive accuracy* is the accuracy that could be expected if this model was used to predict a sequence of sleep stages and their durations. The models used were built to capture variability in sleep architecture as opposed to prediction of the next stage, therefore *predictive error* gives a better measure model quality.

#### Parameter fitting, generalizability, and significance testing

All variables in the model were discrete, and therefore the parameters for each variable were the probability of each possible value of the variable, conditional on each possible value of its parents. This means that parameters in the models associated with stage identity represent transition probabilities (of various order), and parameters associated with stage duration were duration distributions. For example, if the identity of a stage is not influenced by any other variables, then there are 5 parameters representing the probability of transitioning to each stage from any stage (0^th^ Order Transition Probabilities). If the previous stage influences the current stage, there are 5 x 5 parameters, representing the probability of the current stage given each possible previous stage (1^st^ Order Transition probabilities, Table 1).

For analysis of stage durations, we were more interested in the change of the expected duration (a single value per stage) rather than how the distribution changes (4 values per stage, see *Discretization* section). For each stage, we converted the 4-parameter duration distribution back to a single expected (≈mean) duration by taking the dot product with the midpoints of that stages’ discretization bins (as in S3 Table).

To capture the generalizability of our model, we ran every model 14 times, each time removing (and then replacing) one of the datasets from the full set of datasets used to train the model. The parameter estimates from each of these runs were noted, and, similar to multi-level-modeling, we gained a low-resolution distribution of the expected parameter estimates in the population. Parameter values reported in the paper are the mean of these distributions, and the standard deviations are shown as error bars. Additionally, we calculated the *out-of-dataset predictive accuracy* which quantifies the expected accuracy for a new subject from a new dataset (as opposed to estimates for new subject within a dataset). This was achieved using that same k-fold technique as in 2.2.3, where each fold was a dataset.

To test parameter significance, we use a standard bootstrapping technique to generate confidence intervals. Exact confidence intervals, as well as a thorough description of the bootstrapping method used, appear in supplementary methods (S4 and S5 Tables). However, due to the large amount of data used in this study, statistical power increased to the point where trivially small effect sizes led to significance. Instead of focusing on significance, effect sizes were the primary statistic of interest in this paper. The Bayesian Network technique allows us to build in existing knowledge in the form of priors for model parameters. Due to the exploratory nature of this work, we chose uninformed priors for all model parameters so as not to add any specific bias to our model. Note that choice of priors is unlikely to have any influence because of the large amount of data used.

#### Discretization

To train our models, we broke the record of sleep for each subject into a set of data points, one for each bout of a sleep stage. Each data-point contained the stage *duration*, stage *identity*, stage start time (*Time of Day*), time since the start of sleep (*Time Slept*), Sex, Age and BMI of the subject, as well as durations and identities of previous stage. We refer to the current stage as *t* and previous stages as t-1 (one back), t-2 (two back), t-3 (three back). A thorough explanation of this process appears in S2 Text. To simplify modeling, the hierarchical structure of this data (i.e., variables created by the sliding window are ‘nested’ within the same *BMI*, *Sex* and *Age*) was not accounted for in the model.

All variables in our Bayesian network models were discrete valued. By using discrete variable representation, we decreased the complexity of modeling because the exact functional relationship between variables could be undefined. Unfortunately, discretizing a naturally continuous variable (e.g., such as splitting the continuous BMI measure into low BMI and high BMI) also reduced the resolution model predictions. As a first version of this model, we chose simplicity over predictive power and as such, continuous variables were discretized. Furthermore, there is conflict in the field over which parametric functions best describe the distribution of each stage’s durations [22]. Instead of pre-specifying these distributions (as in 28,92), our discretizing scheme essential fits a multinomial distribution to each stage’s duration distribution, which gives a flexible, albeit low resolution, representation.

Where possible, discretization schemes are principled. BMI was split at 25 kg/m^2^, the cutoff for the ‘normal’ category. The age range was split equality into 3 parts, yielding younger (18–42 years), middle age (43–66 years), and older (67–90 years) groups. Duration of all stages closely resembled a log-normal distribution, therefore, durations discretization bins were logarithmically spaced from the minimum duration to the 95^th^ quartile duration (not max, because it would be influenced by outliers. The remaining 5% was included in the last bin). For other variables, such as *Time of Day* and *Time Slept*, discretization could not be made pragmatically. We follow the common approach for Bayesian networks where data is split into quantiles, with an equal number of data points in each discretization bin (3 bins used for each). See S3 Table for exact discretization bin edges.

## Supporting information

S1 Fig Dataset differences between traditional sleep architecture variables. See S2 Table for more information on each dataset. SWS: slow wave sleep; WASO: wake after sleep onset; REM: rapid eye movement sleep.
(TIF)

S2 Fig An example of comparing 4 Bayesian networks. Each defines a different set of hypotheses over the variables considered (not trained on real data). The model with the Bayesian Information Criterion (BIC) score closest to zero (the 3^rd^ model) is most likely to generate the observed data. The K2 algorithm searches across the possible relationships between variables to find the one with the lowest BIC.
(TIF)

S3 Fig Graphical explanation of conversion from an individual’s sleep architecture pattern to data points. SWS: slow wave sleep; WASO: wake after sleep onset.
(TIF)

S1 Table Regression parameters for the relationship between static sleep architecture measures, sex and age after controlling for total sleep time and sleep onset. (DOCX)

S2 Table Data sources. (DOCX)

S3 Table Discretization schemes for continuous variables. (DOCX)

S4 Table Non-significant confidence intervals values from Fig 9 (Model 2A parameters). (DOCX)

S5 Table Non-significant comparisons from Fig 7 (Model 3A parameters). (DOCX)

S1 Text Bootstrapping method for parameter significance. (DOCX)

S2 Text Data parsing scheme. (DOCX)

S3 Text Data availability. (DOCX)
