# Supplementary figures and images for: Quantifying sleep architecture dynamics and individual differences using big data and Bayesian networks
Source: PLoS One. 2018 Apr 11;13(4):e0194604. doi: 10.1371/journal.pone.0194604 (PMC5894981; doi:10.1371/journal.pone.0194604)

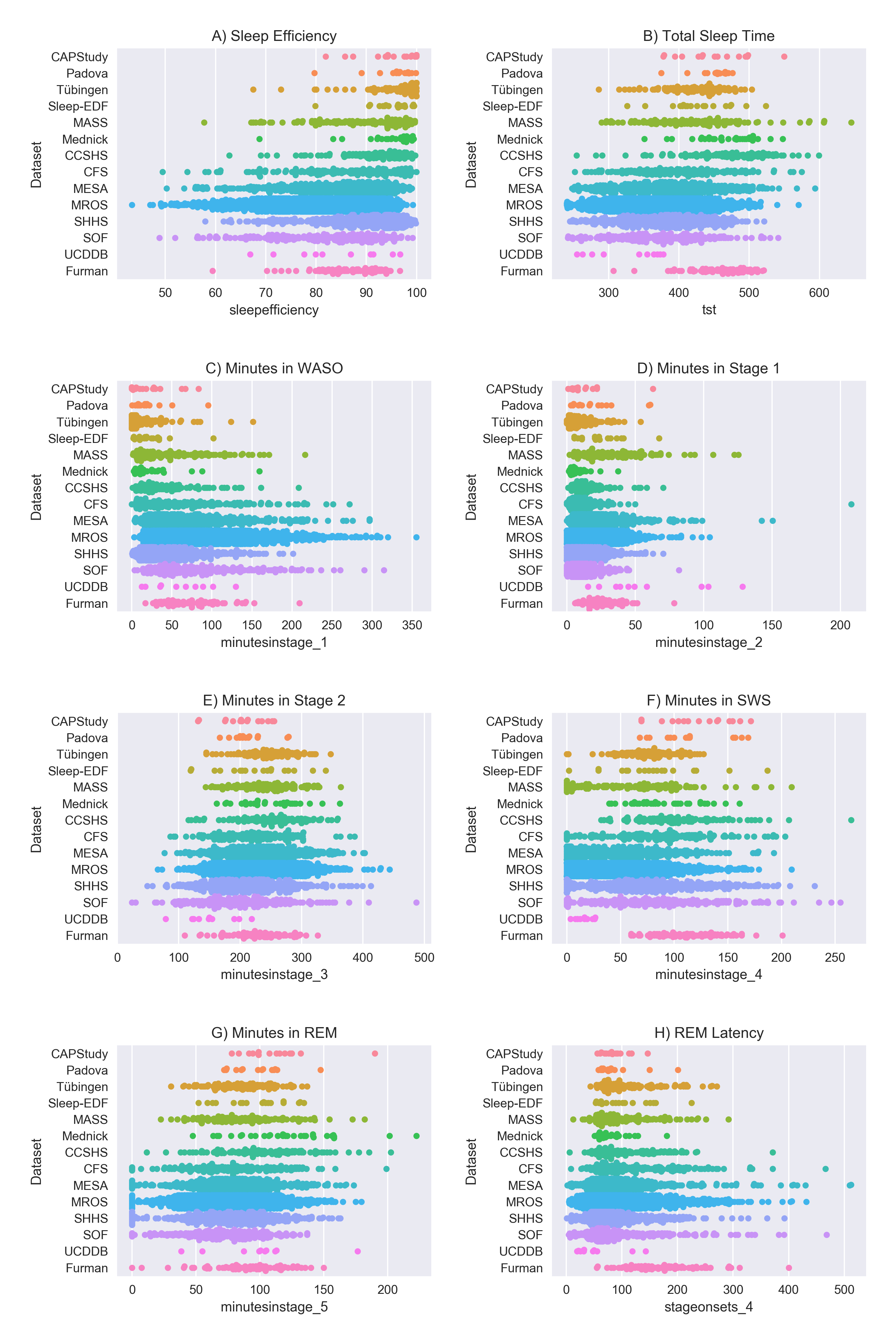

Supplement: S1 Fig — See S2 Table for more information on each dataset. SWS: slow wave sleep; WASO: wake after sleep onset; REM: rapid eye movement sleep. (TIF) [file pone.0194604.s001.tif]

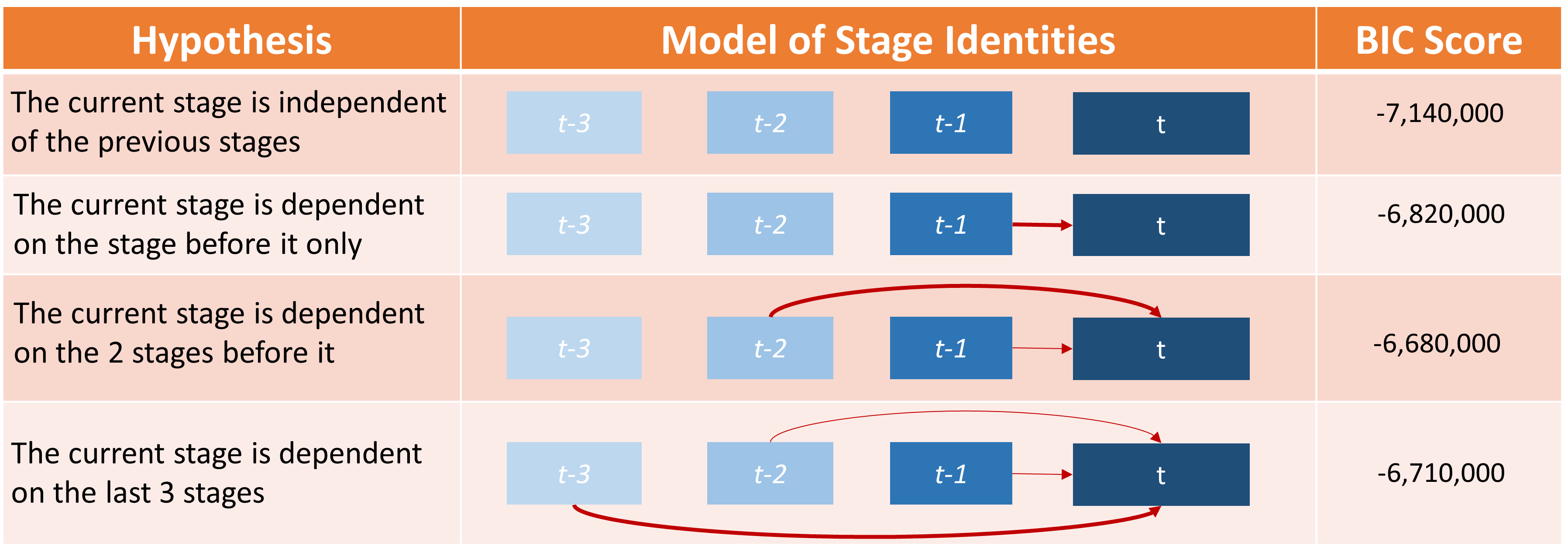

Supplement: S2 Fig — Each defines a different set of hypotheses over the variables considered (not trained on real data). The model with the Bayesian Information Criterion (BIC) score closest to zero (the 3rd model) is most likely to generate the observed data. The K2 algorithm searches across the possible relationships between variables to find the one with the lowest BIC. (TIF) [file pone.0194604.s002.tif]

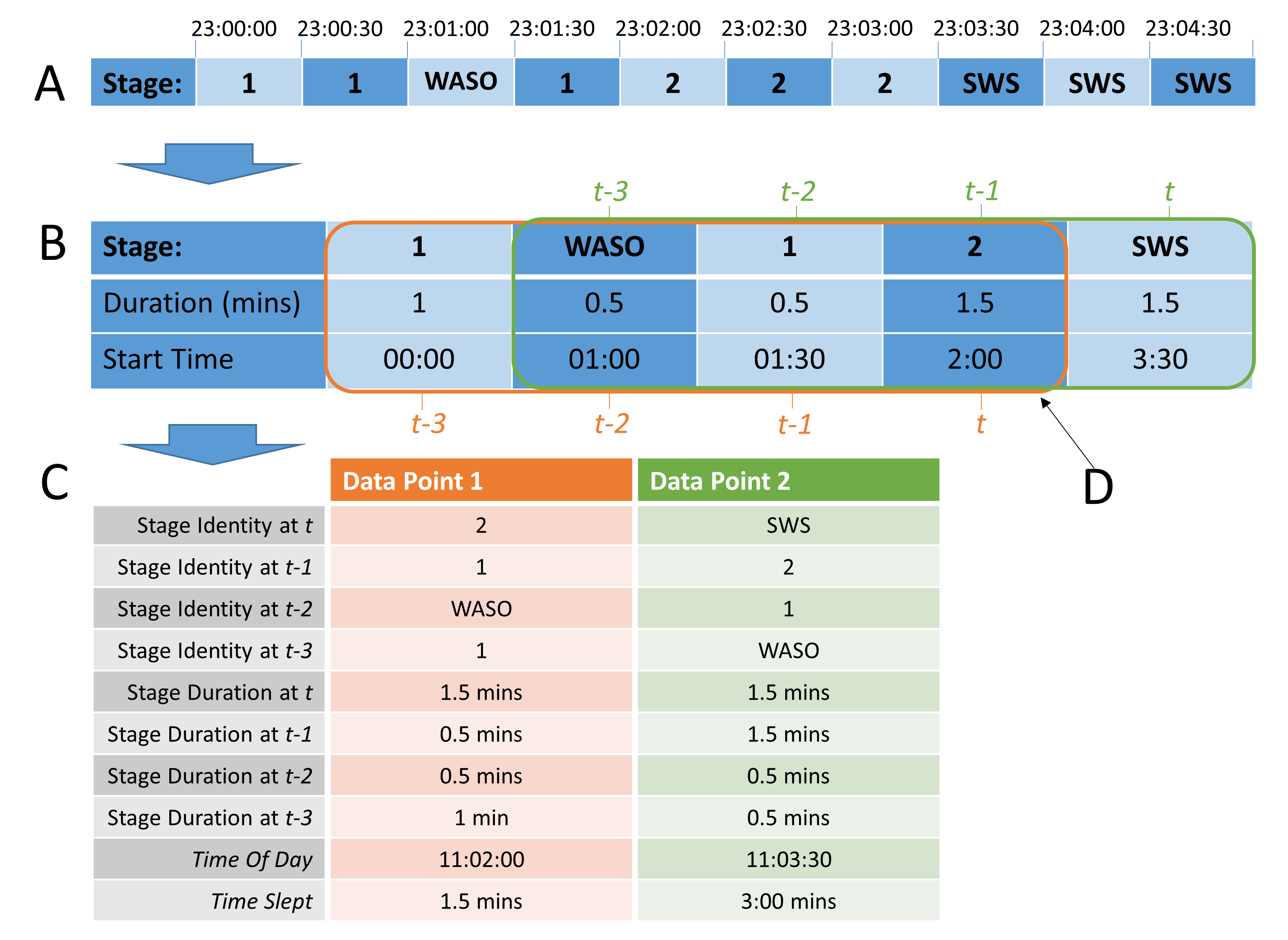

Supplement: S3 Fig — SWS: slow wave sleep; WASO: wake after sleep onset. (TIF) [file pone.0194604.s003.tif]
