# Supplementary material for: Quantifying sleep architecture dynamics and individual differences using big data and Bayesian networks
Source: PLoS One. 2018 Apr 11;13(4):e0194604. doi: 10.1371/journal.pone.0194604 (PMC5894981; doi:10.1371/journal.pone.0194604)
Supplement: S1 Table — (DOCX) [file pone.0194604.s004.docx]

|  | **Sleep Eff** | **WASO** | **Stage 1** | **Stage 2** | **SWS** | **REM** | **REM Latency** |
| --- | --- | --- | --- | --- | --- | --- | --- |
| Mean intercept | 0.92 [0.89,0.95] | 69.69 [49.52,92.96] | 19.10  [8.90, 29.84] | 200.36  [186.78,215.6] | 119.13 [102.79,  136.31] | 58.18 [46.41,  64.86] | 160.62 [140.14,  181.89] |
| Std Dev Intercept | 0.05 [0.03,0.08] | 11.06 [0.33,25.75] | 10.33  [4.42, 16.21] | 17.42  [6.15, 29.72] | 7.58  [0.79, 14.74] | 2.98 [0.16,7.10] | 17.59  [1.03, 34.82] |
| Mean Age | -0.002  [-0.003,-0.001] | -0.94  [-1.84,-0.19] | 0.06  [-0.36, 0.50] | 0.39 [0.09,0.76] | -1.67  [-2.36,-0.91] | 1.10 [0.76,1.53] | -2.02  [-2.66, -1.31] |
| Std Dev Age | 0.001 [0.000,0.001] | 0.25 [0.02,0.54] | 0.14  [0.01, 0.31] | 0.24 [0.02,0.65] | 0.27 [0.03,0.53] | 0.10 [0.02,0.22] | 0.17  [0.01, 0.47] |
| Mean Sex | N/A | N/A | -7.446  [-16.86, 3.003] | -2.67  [-16.20,8.92] | 2.48 [-8.47,13.50] | N/A | -7.04  [-25.33,9.91] |
| Std Dev Age | N/A | N/A | 2.989 [0.141,7.540] | 4.09 [0.12,10.25] | 3.77 [0.20,9.06] | N/A | 5.89  [0.04, 14.41] |
| Mean Age*Sex | N/A | N/A | 0.371  [-0.13,0.89] | 0.26  [-0.05,0.57] | -0.34 [-0.66,-0.07] | N/A | -0.14  [-0.59, 0.25] |
| Std Dev Age*Sex | N/A | N/A | 0.106 [0.008,0.232] | 0.13 [0.00,0.31] | 0.13 [0.01,0.27] | N/A | 0.13 [0.01,0.32] |
| Mean Age^2^ | N/A | 0.02  [0.01,0.02] | 0.000  [-0.004,0.004] | N/A | 0.01 [0.00,0.02] | -0.01  [-0.02,-0.01] | 0.02 [0.01,0.03] |
| Std Dev Age^2^ | N/A | 0.00  [0.00,0.01] | 0.002 [0.000,0.004] | N/A | 0.00 [0.00,0.01] | 0.00 [0.00,0.00] | 0.00 [0.00,0.01] |
| Mean Age^2^*Sex | N/A | N/A | -0.002  [0.008,  0.003] | N/A | N/A | N/A | N/A |
| Std Dev Age^2^*Sex | N/A | N/A | 0.002 [0.000,  0.004] | N/A | N/A | N/A | N/A |
| Mean TST | 0.05 [0.04,0.06] | -17.69 [-22.56,-13.41] | 0.217 [-1.686,1.488] | 34.92 [29.81,39.86] | 8.30 [5.41,11.29] | 19.31 [16.45,21.71] | -3.99 [-8.61,0.68] |
| Std Dev TST | 0.01 [0.01,0.02] | 6.00 [1.99,10.67] | 2.007 [0.113,  4.236] | 7.89 [2.82,14.86] | 3.83 [0.18,9.17] | 4.99 [2.82,7.47] | 5.49 [0.70,11.19] |
| Mean Sleep Onset | 0.02 [0.01,0.03] | -10.01 [-17.24,-2.82] | -0.916  [-2.039,  0.130] | -1.66 [-4.53,1.24] | 1.85 [-1.37,4.27] | 0.51 [-1.46,1.94] | -5.17 [-13.01,2.98] |
| Std Dev Sleep Onset | 0.01 [0.00,0.03] | 10.00 [2.60,16.98] | 0.705 [0.093,  2.199] | 1.77 [0.12,4.47] | 1.76 [0.11,4.97] | 2.16 [0.50,3.80] | 10.67 [1.88,19.33] |

**Notes:** Data from all subjects and across whole night. Mean paramaters values, and standard deviation are at level 2, i.e. mean/std dev across studies. REM: rapid eye movement sleep; SWS: slow wave sleep; WASO: wake after sleep onset; N/A: Non-Applicable.
