# Supplementary material for: Quantifying sleep architecture dynamics and individual differences using big data and Bayesian networks
Source: PLoS One. 2018 Apr 11;13(4):e0194604. doi: 10.1371/journal.pone.0194604 (PMC5894981; doi:10.1371/journal.pone.0194604)
Supplement: S2 Table — (DOCX) [file pone.0194604.s005.docx]

| Dataset | Description | Availability | Number of Records | Mean age (range) | % male |
| --- | --- | --- | --- | --- | --- |
| Mednick Sleep and Cognition Lab[59] | Data collected at University of California, Riverside and University of California, San Diego from an undergraduate population. Scored in house using Rashtscheffen and Kales (R & K).[15] | On request. See S3 Text | 32 | 21 years (18-27) | 53% |
| Sleep Psychophysiology Lab at the University of Padova[70] | Data collected at University of Padova, from an undergraduate population. Scored in house using American Association of Sleep Medicine (AASM) criteria.[88] | On request. See S3 Text | 14 | 25 years (23-28) | 57.1% |
| The Institute of Medical Psychology and Behavioral Neurobiology at the University Tübingen[61–69] | Sleep data collected at the university of Tübingen, Germany in a predominantly college student population. Scored in house using AASM. No BMI information. | On request. See S3 Text | 118 | 23 years (18-35) | 52.5% |
| Furman Sleep Lab[60] | Data collected as Furman University from an undergraduate population. Scored in house using AASM criteria. No BMI information. | On request. See S3 Text | 81 | 20.4 years (18-25) | 50.6% |
| Montreal Archive of sleep studies (MASS)[87] | Open access collaborative database of laboratory-based PSG recordings. Scored using a mixture of R & K and AASM. | www.ceams-carsm.ca/en/MASS | 131 | 45.8 years (18-75 years) | 48.8% |
| MrOS Sleep Study[71–74] | An ancillary study of the Osteoporotic Fractures in Men Study. Men aged 65 and older underwent one full night of PSG. PSG scored at Case Western University Reading Center using modified R & K criteria.[102] | www.sleepdata.org | 1308 | 76.2 years (67.0-90.0) | 100% |
| Cleveland Family Study[71,80,81] | A large family-based study of sleep apnea. PSG Data from Visit 5 used. R & K scoring criteria used. | www.sleepdata.org | 182 | 34 years (18.0-76.6) | 33.5% |
| Study of Osteoporotic Fractures[71,82,83] | Night PSG from a part of a larger multi-center study on to determine risk factors for fractures and falls in older women. Data scored as per MrOS. | www.sleepdata.org | 241 | 82 years (75.0-90.0) | 0% |
| Sleep Heart Health[71,75,76] | Large multi cohort, multi-center study to determine the cardiovascular and other consequences of sleep-disordered breathing. R & K scoring criteria used. | www.sleepdata.org | 446 | 58.6 years (40-87) | 22.6% |
| The Cleveland Children's Sleep and Health Study (CCSHS)[71,77–79] | One of the largest population-based pediatric cohorts. PSG data from visit 3 used. Only subjects over 18 at time of visit used. Scored as per Cleveland Family Study. | www.sleepdata.org | 114 | 18.3 (18-20) | 56.1% |
| Multi-Ethnic Study of Atherosclerosis (MESA)[71] | A collaborative longitudinal investigation of factors associated with the development of subclinical cardiovascular disease in black, white, Hispanic, and Chinese-American men and women. No BMI information. R&K scoring. | www.sleepdata.org | 493 | 67.6 years (55-90) | 36.7% |
| The CAP Sleep Database | A subset of healthy individuals from the 108 subjects in the Cyclic Alternating Pattern sleep database. Scored using R&K. | www.physionet.org/pn4/capslpdb | 16 | 32.4 years (23-42) | 40.0% |
| The sleep-EDF Database[84,85] | A collection of PSG data from two whole night studies (one in a healthy population, and the other a study on the effects of temazepam on sleep). Only the study in health population was used. Modified R&K scoring. | www.physionet.org/pn4/sleep-edfx | 16 | 29 years (25-34) | 50.0% |
| St. Vincent University/University College Dublin Sleep Apnea Database[84] | Overnight recordings from adult subjects with suspected sleep-disordered breathing. Subjects with signs of sleep apnea remove (AHI>15). R&K scoring. | https://physionet.org/pn3/ucddb/ | 10 | 50.1 years (28-65) | 80.0% |
| **Total** |  |  | 3202 | 62.5 years | 60% |

Notes: AHI: Apnea Hypopnea Index; BMI: Body Mass Index.
