# Supplementary material for: Quantifying sleep architecture dynamics and individual differences using big data and Bayesian networks
Source: PLoS One. 2018 Apr 11;13(4):e0194604. doi: 10.1371/journal.pone.0194604 (PMC5894981; doi:10.1371/journal.pone.0194604)
Supplement: S3 Table — (DOCX) [file pone.0194604.s006.docx]

| **Continuous Variable** | **Discretization (Bin Edges)** | **Percentage in Bin** |
| --- | --- | --- |
| *Total Sleep Time* | [0-139.5, 140-269.5, 270-640] mins | 33%, 33%, 33% |
| *Time of Day* | [18:14-00:12, 00:12-02:50, 02:50-10:52] | 33%, 33%, 33% |
| *Duration Wake* | [1-1.5, 2-5, 5.5-18, >18] mins | 40%, 26%, 21%, 13% |
| *Duration Stage 1* | [0-1, 1.5-3, 3.5-9, >9.5] mins |  |
| *Duration Stage 2* | [0-1.5, 2-4.5, 5-15.5, >15.5] mins |  |
| *Duration SWS* | [1-1.5, 2-5, 5.5-18, >18] mins |  |
| *Duration REM* | [1-1.5, 2-5, 5.5-18, >18] mins |  |
| *Duration WASO* | [1-1.5, 2-5, 5.5-18, >18] mins |  |
| *BMI* | [=<25, >25] | 32%, 67% |
| *Age* | [<=42, 43-66, >=67] years | 14%, 19%, 66% |

Notes: Discretization scheme for continuous variables. REM: rapid eye movement sleep; SWS: slow wave sleep; WASO: wake after sleep onset; BMI: Body Mass Index.
