# Supplementary material for: Quantifying sleep architecture dynamics and individual differences using big data and Bayesian networks
Source: PLoS One. 2018 Apr 11;13(4):e0194604. doi: 10.1371/journal.pone.0194604 (PMC5894981; doi:10.1371/journal.pone.0194604)
Supplement: S4 Table — (DOCX) [file pone.0194604.s007.docx]

| **Parameter Type** | **Stage** | **Comparison** | **Difference** | **95%CI** |
| --- | --- | --- | --- | --- |
| 0^th^ Order Trans Prob | Stage 1 | Beginning->Middle | -0.006 | [-0.0068, 0.0065] |
