# Supplementary material for: Quantifying sleep architecture dynamics and individual differences using big data and Bayesian networks
Source: PLoS One. 2018 Apr 11;13(4):e0194604. doi: 10.1371/journal.pone.0194604 (PMC5894981; doi:10.1371/journal.pone.0194604)
Supplement: S5 Table — (DOCX) [file pone.0194604.s008.docx]

| **Outcome Measure** | **Stage** | **Sex** | **Age** | **Time** | **diff** | **95%CI** |
| --- | --- | --- | --- | --- | --- | --- |
| Duration | REM | Female | Old | Middle vs End | 0.031 | [-0.031,0.032] |
| Duration | REM | Male | Old | Middle vs End | 0.031 | [-0.031,0.032] |
| Duration | REM | Female | Old vs Mid | Beginning | -0.008 | [-0.031,0.032] |
| Duration | REM | Male | Old vs Mid | Beginning | -0.008 | [-0.031,0.032] |
| Duration | Stage 2 | Female | Old | Middle vs End | -0.001 | [-0.048,0.05] |
| Duration | Stage 2 | Male | Old | Middle vs End | -0.001 | [-0.048,0.05] |
| Stage Props | REM | Female vs Male | Young | Beginning | -0.003 | [-0.005,0.005] |
| Stage Props | REM | Female vs Male | Young | End | -0.005 | [-0.005,0.005] |
| Stage Props | REM | Female vs Male | Old | Middle | -0.002 | [-0.005,0.005] |
| Stage Props | REM | Female vs Male | Old | End | 0.004 | [-0.005,0.005] |
| Stage Props | REM | Female | Old vs Mid | Beginning | -0.004 | [-0.005,0.005] |
| Stage Props | REM | Female | Mid vs Young | Middle | 0.002 | [-0.005,0.005] |
| Stage Props | Stage 1 | Male | Old | Beginning vs Middle | 0.000 | [-0.003,0.002] |
| Stage Props | Stage 1 | Female vs Male | Young | Middle | 0.000 | [-0.003,0.002] |
| Stage Props | Stage 1 | Female vs Male | Old | Beginning | 0.003 | [-0.003,0.002] |
| Stage Props | Stage 1 | Female | Old vs Mid | Beginning | 0.002 | [-0.003,0.002] |
| Stage Props | Stage 1 | Female | Old vs Mid | Middle | 0.002 | [-0.003,0.002] |
| Stage Props | Stage 2 | Female | Mid | Middle vs End | -0.004 | [-0.007,0.008] |
| Stage Props | Stage 2 | Female | Old | Middle vs End | 0.008 | [-0.007,0.008] |
| Stage Props | Stage 2 | Female vs Male | Mid | End | 0.001 | [-0.007,0.008] |
| Stage Props | Stage 2 | Female | Mid vs Young | Middle | 0.000 | [-0.007,0.008] |
| Stage Props | SWS | Female vs Male | Young | Beginning | 0.003 | [-0.007,0.007] |
| Stage Props | SWS | Female vs Male | Young | End | 0.003 | [-0.007,0.007] |
| Stage Props | SWS | Female | Old vs Young | End | 0.000 | [-0.007,0.007] |
| Stage Props | SWS | Male | Old vs Mid | End | 0.007 | [-0.007,0.007] |
| Stage Props | WASO | Female | Young | Middle vs End | 0.002 | [-0.009,0.009] |
| Stage Props | WASO | Female vs Male | Young | Middle | 0.005 | [-0.009,0.009] |
| Stage Props | WASO | Female vs Male | Young | End | -0.009 | [-0.009,0.009] |
| Stage Props | WASO | Female vs Male | Mid | Beginning | -0.009 | [-0.009,0.009] |
| Stage Props | WASO | Female vs Male | Mid | Middle | -0.008 | [-0.009,0.009] |
| Stage Props | WASO | Female vs Male | Old | Beginning | -0.009 | [-0.009,0.009] |
| Trans Probs | REM | Male | Old | Middle vs End | 0.002 | [-0.003,0.003] |
| Trans Probs | REM | Female vs Male | Young | Beginning | 0.002 | [-0.003,0.003] |
| Trans Probs | REM | Female vs Male | Mid | Beginning | 0.003 | [-0.003,0.003] |
| Trans Probs | REM | Female vs Male | Mid | End | 0.002 | [-0.003,0.003] |
| Trans Probs | Stage 1 | Female | Mid | Beginning vs Middle | 0.005 | [-0.006,0.006] |
| Trans Probs | Stage 1 | Female | Old | Beginning vs Middle | 0.001 | [-0.006,0.006] |
| Trans Probs | Stage 1 | Male | Mid | Beginning vs Middle | -0.001 | [-0.006,0.006] |
| Trans Probs | Stage 1 | Female vs Male | Young | Middle | 0.003 | [-0.006,0.006] |
| Trans Probs | Stage 1 | Male | Old vs Mid | Middle | 0.006 | [-0.006,0.006] |
| Trans Probs | Stage 2 | Female vs Male | Young | Middle | 0.004 | [-0.005,0.005] |
| Trans Probs | Stage 2 | Female vs Male | Old | Beginning | -0.004 | [-0.005,0.005] |
| Trans Probs | Stage 2 | Female | Old vs Mid | Middle | 0.005 | [-0.005,0.005] |
| Trans Probs | Stage 2 | Male | Old vs Mid | End | -0.005 | [-0.005,0.005] |
| Trans Probs | SWS | Male | Young | Beginning vs Middle | -0.005 | [-0.007,0.007] |
| Trans Probs | SWS | Female vs Male | Young | Beginning | 0.003 | [-0.007,0.007] |
| Trans Probs | SWS | Female vs Male | Young | Middle | -0.007 | [-0.007,0.007] |
| Trans Probs | SWS | Female | Old vs Mid | Middle | -0.001 | [-0.007,0.007] |
| Trans Probs | SWS | Male | Old vs Mid | Beginning | 0.004 | [-0.007,0.007] |
| Trans Probs | SWS | Male | Old vs Mid | End | 0.008 | [-0.007,0.007] |
| Trans Probs | WASO | Female vs Male | Young | Beginning | -0.007 | [-0.006,0.006] |
| Trans Probs | WASO | Female vs Male | Young | Middle | -0.006 | [-0.006,0.006] |
| Trans Probs | WASO | Female vs Male | Mid | End | -0.007 | [-0.006,0.006] |
| Trans Probs | WASO | Female | Old vs Mid | Middle | 0.001 | [-0.006,0.006] |
| Trans Probs | WASO | Female | Old vs Mid | End | -0.005 | [-0.006,0.006] |

**Notes**: REM: rapid eye movement sleep; SWS: slow wave sleep; WASO: wake after sleep onset.
