# Supplementary material for: Quantifying sleep architecture dynamics and individual differences using big data and Bayesian networks
Source: PLoS One. 2018 Apr 11;13(4):e0194604. doi: 10.1371/journal.pone.0194604 (PMC5894981; doi:10.1371/journal.pone.0194604)
Supplement: S1 Text — (DOCX) [file pone.0194604.s009.docx]

**S1 Text: Bootstrapping method for parameter significance**

After the K2 algorithm found the best fitting model by comparing BIC scores, we tested if model parameters significantly differed between groups using a standard bootstrapping approach. First, we randomly sampled each data record, with replacement, from the full set of records. We then split these into two groups and calculated parameters associated with our variables of interest for each group (for example, 1^st^ order transition probabilities). Next, we took the difference of these parameters and saved this sample, this gives a single sample of the expected difference between these parameters due to chance. Repeating the above 3 steps, 2000 times, gave us a distribution of the expected difference of parameters values due to chance, from which a 95% confidence interval was calculated. We then compared our actual parameter difference between groups of interest (e.g. difference in 1^st^ order transition probabilities parameters between male and female or between start of the night and end of the night, etc.) to this 95% confidence interval, and if it was outside this interval, the parameter difference was considered statistically significant (i.e., the grouping variable had an effect on the parameter values).
