# Supplementary material for: Quantifying sleep architecture dynamics and individual differences using big data and Bayesian networks
Source: PLoS One. 2018 Apr 11;13(4):e0194604. doi: 10.1371/journal.pone.0194604 (PMC5894981; doi:10.1371/journal.pone.0194604)
Supplement: S2 Text — (DOCX) [file pone.0194604.s010.docx]

**S2 Text:** **Data Parsing Scheme**

Each record of sleep was converted into a set of data points to train our models. We started with the raw sleep architecture data, which gave the identity of the sleep stage every 30 seconds. We then removed the final wake period and the wake before sleep onset. All other wake (WASO) was included. The raw data was then converted into stage identity and stage duration vectors, with one element for each bout of a stage (**S3 Fig**, A to B). Next, to slice the stage identity and duration vectors into data points, a 4-element sliding window was passed sequentially over each vector to turn blocks of 4 consecutive elements into a single data-point of 8 variables (4 stage identify variables + 4 corresponding stage duration variables) (**S3 Fig**, B to C). The stage identity and duration in the leading positon of this sliding window was marked *t*, the stage 1 back from that was marked t-1 (**S3 Fig**, D), etc. We will refer to the stage t as the current stage. The start time of the current stage as a fraction of the 24hr clock (*Time of Day* - zero at midnight, time before midnight as negative) and the total amount of time spent asleep up to that point (*Time Slept*) were also calculated. Finally, *BMI, Sex* and *Age* for that participant were added, giving a final set of 5+8 variables per data point used for model fitting. Note that when less than 4 previous stages were considered, the number of variables extracted from the stage identify and duration vectors was reduced accordingly.
