# Supplementary material for: Quantifying sleep architecture dynamics and individual differences using big data and Bayesian networks
Source: PLoS One. 2018 Apr 11;13(4):e0194604. doi: 10.1371/journal.pone.0194604 (PMC5894981; doi:10.1371/journal.pone.0194604)
Supplement: S3 Text — (DOCX) [file pone.0194604.s011.docx]

**S3 Text: Data availability**

The majority of data used in the current study is open access and can be directly downloaded or requested through each dataset’s corresponding website (see S2 Table). Data from Mednick Sleep and Cognition Lab (contact: Lexus Hernandez, lthernan@uci.edu), Sleep Psychophysiology Lab (contact: Lexus Hernandez, lthernan@uci.edu), The Institute of Medical Psychology and Behavioral Neurobiology at the University Tübingen (contact: Susanne Diekelmann, susanne.diekelmann@uni-tuebingen.de) and the Furman Sleep Lab (contact: Erin Wamsley, erin.wamsley@furman.edu) were collected with a consent form which did not specify open access sharing. However, a clause allows this data to be shared with collaborators in the same manner as the authors, and any interested parties should contact the corresponding data owners as referenced above. The authors did not have any special access privileges to these data.
